# Non-cell autonomous astrocyte-mediated neuronal toxicity in prion diseases

**DOI:** 10.1186/s40478-021-01123-8

**Published:** 2021-02-05

**Authors:** Rajesh Kushwaha, Anshuman Sinha, Natallia Makarava, Kara Molesworth, Ilia V. Baskakov

**Affiliations:** 1 Center for Biomedical Engineering and Technology, University of Maryland School of Medicine, 111 S. Penn St, Baltimore, MD 21201 USA; 2 Department of Anatomy and Neurobiology, University of Maryland School of Medicine, Baltimore, MD 21201 USA

**Keywords:** Prions, Prion diseases, Astrocytes, Microglia, Neuroinflammation, Synaptic toxicity

## Abstract

Under normal conditions, astrocytes perform a number of important physiological functions centered around neuronal support and synapse maintenance. In neurodegenerative diseases including Alzheimer’s, Parkinson’s and prion diseases, astrocytes acquire reactive phenotypes, which are sustained throughout the disease progression. It is not known whether in the reactive states associated with prion diseases, astrocytes lose their ability to perform physiological functions and whether the reactive states are neurotoxic or, on the contrary, neuroprotective. The current work addresses these questions by testing the effects of reactive astrocytes isolated from prion-infected C57BL/6J mice on primary neuronal cultures. We found that astrocytes isolated at the clinical stage of the disease exhibited reactive, pro-inflammatory phenotype, which also showed downregulation of genes involved in neurogenic and synaptogenic functions. In astrocyte-neuron co-cultures, astrocytes from prion-infected animals impaired neuronal growth, dendritic spine development and synapse maturation. Toward examining the role of factors secreted by reactive astrocytes, astrocyte-conditioned media was found to have detrimental effects on neuronal viability and synaptogenic functions via impairing synapse integrity, and by reducing spine size and density. Reactive microglia isolated from prion-infected animals were found to induce phenotypic changes in primary astrocytes reminiscent to those observed in prion-infected mice. In particular, astrocytes cultured with reactive microglia-conditioned media displayed hypertrophic morphology and a downregulation of genes involved in neurogenic and synaptogenic functions. In summary, the current study provided experimental support toward the non-cell autonomous mechanisms behind neurotoxicity in prion diseases and demonstrated that the astrocyte reactive phenotype associated with prion diseases is synaptotoxic.

## Introduction

Chronic neuroinflammation is a major feature of neurodegenerative diseases including Alzheimer’s, Parkinson’s and prion diseases [1]. The main manifestation of chronic neuroinflammation is a sustained transformation of homeostatic phenotype of microglia and astrocytes into reactive phenotypes [2, 3]. Transgenic mouse models of human neurodegenerative diseases advanced our understanding of diversity of glial reactive phenotypes [4–6]. However, the question of whether animal models faithfully recapitulate chronic neuroinflammation associated with diseases in humans has been raised on multiple occasions [7–9]. Unlike modeling of most neurodegenerative diseases that rely on transgenic animals, transmissible bona fide prion diseases can be induced in wild-type or inbred animals upon infection with the disease-associated form of the prion protein, or PrP^Sc^ [10].

Prion disease displays a surprisingly diverse range of molecular and clinical phenotypes [11]. The diversity of disease phenotypes is attributed to the structural diversity of PrP^Sc^ states referred to as prion strains [12]. Posttranslation modifications appear to play an important role in dictating strain-specific disease phenotype. The cellular form of the prion protein, or PrP^C^, is post-translationally modified with the glycosylphosphatidylinositol (GPI) anchor and one or two sialylated N-linked glycans [13–16]. Among hundreds of PrP^C^ sialoglycoforms expressed by a cell, individual prion strains selectively recruit those sialoglycoforms that can be accommodated within a strain-specific structure [17–19]. As a result of selective recruitment, unique strain-specific patterns of carbohydrate epitopes is formed on PrP^Sc^ surface dictating strain-specific diseases phenotypes [20, 21].

Among the possible mechanisms responsible for loss of neuronal functions is a direct toxic signaling triggered by PrP^Sc^ on the surface of neuronal cells, either via PrP^C^-dependent or independent pathways. Exploring the mechanisms of direct toxicity of PrP^Sc^ has been the focus for the last 3 decades and has received solid experimental support [12, 22–33]. Yet, it is challenging to explain how PrP^Sc^ strains that colocalize predominantly with astrocytes induce neuronal death [34].

Astrocytes can replicate and accumulate PrP^Sc^ independently of neurons [35–38]. Astrocytes were found to accumulate prions early, ahead of neuropathological changes involving vacuolation or neuronal loss [39]. Expression of PrP^C^ exclusively in astrocytes was found to be sufficient for supporting prion replication and inducing lethal prion diseases [35, 40, 41]. Finally, the fact that PrP^Sc^ strains that colocalize with astrocytes or microglia exhibit short incubation times to disease raises the possibility that alternative, non-cell autonomous mechanisms exist [21].

The majority of previous studies on neuroinflammation in prion diseases focused on reactive microglia [reviewed in [42, 43]], whereas considerably less is known about the role of reactive astrocytes [reviewed in [44]]. Historically, studies of the astrocytic response in prion diseases primarily focused on morphological changes associated with reactive astrogliosis and analyses of expression of glial fibrillary acidic protein (GFAP) or its transcripts [45–48]. However, recent studies shed a new light on astrocyte dysfunction, suggesting that a possible new mechanism might contribute to neuronal death in prion diseases [49, 50]. Activation of unfolded protein response in primary astrocytes was found to induce the reactivity states with deficient synaptogenic function [49]. Region-specific analysis of gene expression revealed that the astrocytes responded to prion infection much earlier than neurons, and perhaps even sooner than microglia [50]. However, it is not known whether upon activation, astrocytes acquire neuroprotective or neurotoxic phenotypes. Recent studies illustrated that highly infectious prions are not directly neurotoxic [51], raising a possibility that mechanisms alternative to the direct toxicity of PrP^Sc^ to neurons exists.

Under healthy conditions, astrocytes perform several important physiological functions including support of neuronal growth, modulation of neurotransmission, formation and maintenance of synapses, regulation of blood flow, supplying energy and providing metabolic support to neurons, maintaining the blood–brain-barrier and more [52–54]. It is not known whether astrocytes lose their ability to perform normal physiological functions in reactive states. It is also not known, whether in the reactive state, astrocytes are neurotoxic or neuroprotective. The current work addresses this question by testing direct effects of reactive astrocytes isolated from prion-infected mice on primary neuronal cultures. The current work provides experimental support toward the non-cell autonomous mechanisms of neurotoxicity in prion diseases and demonstrates that the astrocyte reactive phenotype associated with prion diseases is synaptotoxic.

## Materials and methods

### Reagents and kits

Poly-L-lysine (PLL), Poly-D-lysine (PDL), sodium bicarbonate, tween 20, protease inhibitor cocktail (PIC), paraformaldehyde (PFA), bovine serum albumin (BSA), normal goat serum (NGS), horse serum, CellLytic MT mammalian cell lysis buffer, triton-X-100, ponceau S and dimethyl sulfoxide (DMSO) were procured from Sigma Chemical Co. (St. Louis, MO). Trypsin–EDTA, dulbecco’s modified eagle medium: F12 (DMEM/F12), neurobasal medium, B27 supplement, N2 supplement, trypsin inhibitor, Hank’s balanced salt solution (HBSS), phosphate buffer saline (PBS), dulbecco’s phosphate buffered saline (DPBS), antibiotic–antimycotic, fetal bovine serum (FBS), glutamax and protein ladder were purchased from Invitrogen (Carlsbad, CA). Adult mouse brain dissociation kit, myelin removal solution, cell debris removal solution, RBC lysis solution, LS column and C-tubes were from Miltenyi Biotec (Bergisch Gladbach, Germany). Papain dissociation kit was from Worthington Biochemical Corporation (Lakewood, NJ). VECTASHIELD mounting medium with DAPI was from Vector Laboratories (Burlingame, CA) and SuperSignal West Femto Maximum Sensitivity Substrate was purchased from Thermo Scientific (Rockford, IL). Aurum Total RNA Mini Kit, SYBR Green and iScript cDNA Synthesis Kit were procured from Bio-Rad laboratories (Hercules, CA). The ELISA kit for IL-1a, TNFa and IL-6 was purchased from R&D Systems (Minneapolis, MN) and C1q from LSBio (Cambridge, UK). Bicinchoninic Acid (BCA) protein assay kit, 70 μm nylon mesh filter, 0.22 µm filter, MTT (3-(4,5-dimethylthiazol-2-yl)-2,5-diphenyltetrazolium bromide), polyvinylidenefluoride (PVDF) membrane were procured from Millipore (Temecula, CA).

### Antibodies

Chicken polyclonal antibody to GFAP (cat. AB5541), mouse monoclonal antibodies to β-actin (cat. A5441), horseradish peroxidase (HRP) conjugated secondary anti-rabbit IgG (cat. A0545) and anti-mouse IgG (cat. A9044) were procured from Sigma-Aldrich (St. Louis, MO). Rabbit polyclonal antibody to Iba1 (cat. 01919741) was from Wako (Richmond, VA). Mouse monoclonal antibody to PSD-95 (cat. 75028020) was from Antibodies Incorporated (Davis, CA). Rabbit monoclonal antibodies to synaptophysin (cat. ab32594), Drebrin (cat. ab11068), S100β (cat. ab52642), Olig2 (cat. ab136253), CD11b (cat. ab133357) and chicken monoclonal antibody to MAP2 (cat. ab4542) were from Abcam (Cambridge, MA). Rabbit monoclonal antibody to GFAP (cat. 12389) was from Cell Signaling Technology (Danvers, MA). Mouse monoclonal antibody to NeuN (cat. mab377) and rabbit polyclonal antibody to LCN2 (cat. AB2267) were from Millipore (Temecula, CA). Anti-ACSA-2 MicroBead Kit (cat. 130–097-678) was from Miltenyi Biotec (Bergisch Gladbach, Germany). Alexa Fluor 488 goat anti-rabbit IgG, Alexa Fluor 488 goat anti-chicken IgG, Alexa Fluor 488 goat anti-mouse IgG, Alexa Fluor 546 goat anti-rabbit IgG, Alexa Fluor 546 goat anti-mouse IgG and Alexa Fluor 647 goat anti-mouse IgG secondary antibodies were from Invitrogen (Carlsbad, CA).

### Animals

C57BL/6J male mice were housed in a 12 h day-and-night cycle environment with ad libitum availability of diet and water. Six-week-old C57BL/6J male mice were intraperitoneally inoculated with 200 µl volume of 1% 22L brain homogenate in PBS under anesthetic conditions. The age-matched male control groups were intraperitoneally injected with PBS (200 µl volume) only. Animals were regularly observed and scored for the neurological signs and disease progression. The first clinical signs were observed at 155–190 days post-inoculation (dpi) and involved hind-limb clasping, ataxia and loss of weight. Mice were scored and euthanized when they showed consistent progression of the diseases (developed kyphosis, became lethargic or could no longer ambulate) at 176–238 days post-inoculation. Upon euthanasia, brains were dissected out and cortexes were used for primary cultures.

### Adult primary astrocyte culture

Adult primary cortical astrocyte cultures were prepared using 75 clinically sick 22L-infected and 75 age-matched control mice according to the following protocol. Using micro dissecting scissors, the skin was opened at the midline of the head, skull was cut at the midline fissure and brain was released from the skull cavity. One brain was used per individual culture. A brain was gently transferred to a 60 mm petri dish and was rinsed with cold DPBS to remove adhering blood. After removal of meninges, the brain cortices were dissociated and digested using papain-based enzymes dissociation solution and incubated on the gentleMACS octo dissociator system for 30 min per manufacturer’s instruction (Miltenyi Biotec). Following digestion, cells were re-suspended in buffer containing DPBS, 100 U/ml penicillin and 100 µg/ml streptomycin, then non-cellular debris was removed by passing the cell suspension through 70 µm nylon single-cell strainer. The clear suspension was then centrifuged for 10 min at 1000 rpm. The obtained pellet was incubated with myelin removal solution in 4 °C for 10 min and centrifuged at 1000 rpm for 5 min. The pellet obtained was re-suspended in complete astrocyte growth media [DMEM/F12 (containing 365 µg/ml L-glutamine, 1 mM sodium pyruvate), 10% heat inactivated FBS, 100 U/ml penicillin and 100 µg/ml streptomycin]. Cells were then seeded onto poly-L-lysine (PLL)-coated chamber slides/cover slips or culture flasks at plating density 3–4 × 10^4^ per well or 7 × 10^5^ per flask and grown in a humidified CO_2_ incubator at 37ºC with 5% CO_2_. The next day after plating the cells, complete media was changed to remove debris and unattached dead cells. After incubating primary astrocyte cultures for 6–7 days, medium was replaced completely. Flasks were wrapped in plastic, placed on a shaker platform in a horizontal position with the medium covering the cells, and were shaken at 150 rpm for 1 h at 37º C to separate oligodendrocytes and microglia from the culture. Immediately after shaking, astrocyte-enriched cultures were washed with PBS, fresh astrocyte growth culture medium was added and cultured until confluence (2–3 weeks). Culture medium was replaced every 2 days.

### Acute astrocyte isolation

Acute isolation of astrocytes from adult C57BL/6J mice were performed according to previously described protocol [55]. First, an Adult Brain Dissociation Kit (cat. 130-107-677, Miltenyi Biotec) was used to dissociate cortical brain tissue into single-cell suspensions according to the manufacturer’s instructions. Adult mice were sacrificed under anesthesia with the brain quickly removed and stored in cold DPBS. Cortical tissue was dissected out (one brain per isolation) and kept in cold DPBS. Meninges were carefully removed, cortical tissue were minced and then transferred into gentleMACS C tubes (cat.130-093-237) which contained 1950 µL of enzyme mixture 1 solution and 30 µL of enzyme mixture 2 solution. The tightly closed C tubes were then incubated onto rotated gentleMACS Octo Dissociator with Heater for tissue dissociation (30 min). The obtained dissociated cells were re-suspended in DPBS, filtered with MACS SmartStrainer (70 µm) to remove any non-dissociated tissue and centrifuged for 10 min at 1000 rpm. The obtained pellets were re-suspended in cold DPBS and cell suspension was subjected to debris removal and red blood cell removal. The single cell-suspension obtained was then subjected to magnetic labeling with an Anti-ACSA-2 MicroBead Kit (cat. 130-097-678, Miltenyi Biotec) according to the manufacturer’s instructions. The cell pellets re-suspend were re-suspend in buffer and the cell suspension was blocked with 10–15 µL of FcR blocking reagent at 4 °C for 10 min. 10 µL of Anti-ACSA-2 MicroBeads per 10^7^ total cells was added in cell suspension, mixed well and incubated for 15 min at 4 °C in the dark. The cells were washed by adding 2 mL of AstroMACS separation buffer (cat. 130–117-336, Miltenyi Biotec) and centrifuged at 1500 rpm for 5 min to remove excess beads from the solution. The obtained pellets were re-suspended with buffer and loaded onto an MS Column (cat. 130-042-201, Miltenyi Biotec), which was placed in the magnetic field of a MACS magnetic cell separator. The column was washed with 3 mL buffer to remove unlabeled cells and the magnetically labeled ACSA-2-positive astrocytes cells were eluted as the positively selected cell fraction buffer after removing the column from the magnet separator. Then, RNA was isolated from purified astrocytes for Nanostring analysis.

### Primary neuronal culture

Primary neuronal culture was prepared according to the previously described protocol [56]. Cerebral cortices of post-natal day-1 (PND-1) pups (4–5 brains per preparations) were dissected out in Earle’s balanced salt solution (EBSS). The outermost thin layer of meninges was removed carefully and tissues were digested using Papain dissociation kit according to the manufacturer’s instruction (Worthington Biochemical Corporation). Gently minced cortical tissue was incubated with the Papain-DNase solution (20 units/ml papain and 0.005% DNase) for 30 min in humidified CO2 incubator at 37 °C. The mixture was triturated with 5 ml pipette; cloudy cell suspension was carefully removed, placed in sterile screw capped tube and centrifuged at 1000 rpm for 5 min at room temperature (RT). The pellet obtained was re-suspended in DNase added albumin-inhibitor solution and a discontinuous density gradient solution was prepared through an overlay of cell suspension onto the albumin-inhibitor solution and centrifuged at 700 rpm for 6 min at RT. The supernatant containing non-cellular debris was discarded and pelleted cells were re-suspended in neurobasal medium containing B-27 supplement (1X), L-glutamine (0.5 mM), penicillin (100 U/ml), streptomycin (100 µg/ml) and 1% heat inactivated horse serum. Cells were seeded on poly-D-lysine (PDL) and laminin-coated cultured flask or chamber slide/coverslip at a plating density of 7 × 10^5^ cells per flask and 3–4 × 10^4^ cells per well of chamber slide and grown in a humidified CO_2_ incubator at 37 °C. Cytosine arabinoside (2.5 μM) was added to the cultures on the second day after plating to inhibit the proliferation of non-neuronal cells. Half of the culture medium was changed every 2 days. These cultures were maintained for up to 12–14 days in vitro (DIV) to allow for the development and maturation of dendritic synapses and spines. This protocol produced a neuron-enriched culture and 90–95% of cells were co-stained with the NeuN and MAP2.

### Primary microglia culture

Adult primary microglia cultures were prepared using clinically sick 22L-infected or age-matched control mice as previously described with the minor modifications [57]. Cortical tissue was dissected out (one brain per culture) and kept in cold DPBS. Meninges were carefully peeled from the cortices, then the tissues were transferred to an enzymes cocktail prepared using adult mouse brain dissociation kit (Miltenyi Biotec) and incubated onto rotated gentleMACS octo dissociator system for 30 min, according to manufacturer’s instruction. Digested cells were re-suspended in DPBS and filtered through a 70 μm single-cell strainer followed by centrifugation at 1000 rpm at 4 °C for 10 min. The pellets obtained were re-suspended in 30% isotonic Percoll (GE Healthcare) and PBS (1X) was gently overlaid on the cell suspension. The Percoll gradient solution was centrifuged at 4000 rpm 4 °C for 30 min without breaks. The upper portion (debris and supernatant) containing myelin and astrocytes was carefully aspirated. For removing remaining red blood cells (RBC), the pellet was dissolved in RBC lysis buffer and incubated at 4 °C for 6–8 min, then the solution was centrifuged at 2000 rpm for 5 min. The pellet was re-suspended in growth media [DMEM/F12 (containing 365 µg/ml L-glutamine, 1 mM sodium pyruvate), 10% heat inactivated FBS, 100 U/ml penicillin and 100 µg/ml streptomycin]. Cells were then seeded onto PDL-coated chamber slides or culture flasks at plating density of 3–4 × 10^4^ per well or 7 × 10^5^ cells per flask and grown in a humidified CO_2_ incubator at 37º C with 5% CO_2_ until confluence (10–20 days). Culture medium was replaced every 2 days. The purity of microglial cultures was confirmed by co-immunostaining of CD11b (microglia marker) with GFAP, NeuN or Olig2.

### Primary neuron-astrocytes co-culture

Neuron-astrocyte co-cultures were prepared by plating neurons onto the astrocyte-feeder layer, as previously described [58]. Briefly, primary astrocytes isolated from clinically sick 22L-infected mice or age-matched controls were grown on PDL and laminin coated coverslips/chamber slides for 1 week, then cultures were treated with cytosine arabinoside (2.5 μM) to arrest the growth of dividing cells. A day prior to plating neuronal cells (9–10 DIV), media was replaced with the fresh co-culture media containing 50% astrocytic and 50% neuronal growth media. Cultures were kept in the CO2 incubator for 1 day. Cell suspension of primary neurons (approx. 20,000 per well) was then added to the astrocytes on the coverslips/chamber slides and grown in co-culture media for another 10–12 DIV. Half of the culture medium was changed every 2 days.

### Preparation of conditioned medium

To obtain astrocyte-conditioned media (ACM), primary astrocytes were plated at a density of 7 × 10^5^ per culture flask and grown in a humidified CO_2_ incubator at 37ºC as describe above. After achieving 50–60% confluency, the monolayes of astrocytes were subjected to a neuronal growth media to generate ACM. Media was replenished every 2 days. After 70–80% confluency (2–3 weeks), media was collected and centrifuged at 1000 rpm for 5 min to remove cellular debris and used immediately. To obtain microglia-conditioned media (MCM), primary microglia were plated at a density of 7 × 10^5^ per culture flask and grown in a humidified CO_2_ incubator. After 70–80% confluency (2–3 weeks), media was collected and centrifuged at 1000 rpm for 5 min to remove cellular debris and used immediately.

### Cell viability assay

An MTT (3-(4,5-dimethylthiazol-2-yl)-2,5-diphenyltetrazolium bromide) assay was performed according to the manufacturer protocol. Primary neuronal cells were seeded in PDL-laminin coated 96-well culture plates at a density of 1 × 10^4^ cells per well in triplicates and grown until 70–80% confluence. After incubation of neuronal cells with 22L or control astrocyte-conditioned medium (22L-ACM or CT-ACM, respectively) for the indicated time periods, the culture mediums were cautiously aspirated, and incubated with the MTT reagent at 37 °C for 4 h in a 5% CO2 in the culture incubator. Three wells that contained only medium were used as blank for absorbance reading. 100 μl of detergent reagent was added to all wells and the plate was kept for 2 h in the dark at room temperature. Absorbance was determined at 570 nm using a Microplate reader (Tecan, Infinite 200 PRO, Switzerland). The cell viability was expressed as a percentage normalized relative to the viability in CT-ACM-treated cells. Three independent experiments, each in triplicate, were performed using ACMs from cultures originating from three individual animals per condition.

### Enzyme-linked immunosorbent assay (ELISA)

The levels of secreted C1q, TNFa and IL-1a in microglia conditioned medium (MCM) and IL-6 in ACMs were determined using the ELISA kit according to manufacturer’s instruction. Briefly, primary astrocytes or microglia were plated at a density of 7 × 10^5^ per culture flasks and grown in a humidified CO_2_ incubator at 37 ºC. Conditioned media was collected as describe above, then concentration of C1q, TNFa and IL-1a in MCM, and concentration of IL-6 in ACM were quantified by ELISA using mouse C1q, TNFa, IL-1a and IL-6 kits, respectively. Three independent experiments analysed ACMs or MCMs collected from three individual cultures, each in triplicate.

### RT-qPCR

70–80% confluence astrocyte, microglia or neuronal cultures grown in 6-well culture plates (3.0 × 10^5^ cells) were washed with PBS followed with the isolation of total RNA using the Aurum Total RNA Mini Kit (Bio Rad, Hercules, CA) according to manufacturer’s instruction. Total RNA was dissolved in elution buffer and then, the quantity and purity of mRNA were determined using the NanoDrop ND-1000 Spectrophotometer (Thermo Fisher Scientific, Waltham, MA). Complementary DNA (cDNA) synthesis was performed using the iScript cDNA Synthesis Kit as described elsewhere. The cDNA was amplified with a CFX96 Touch Real-Time PCR Detection System (Bio-Rad, Hercules, CA) using SsoAdvanced Universal SYBR Green Supermix. The PCR protocol consisted of incubation at 95 °C for 2 min followed by 40 amplification cycles at 95 °C for 5 s and 60 °C for 30 s. Optimum primer pairs for genes of interest and housekeeping gene, glyceraldehydes 3-phosphate dehydrogenase (*Gapdh*) were designed using Primer Express version 2.0.0 (Table 1). The ΔCt for each RNA sample was calculated by subtracting the mean Ct of housekeeping gene, *Gapdh* from the mean Ct of the gene of interest and then relative mRNA gene expression was calculated using 2^−ΔΔCt^ method as described elsewhere. Three independent experiments in triplicate were carried out using three different culture preparations.

**Table 1:** Primer sequences for qRT-PCR

Primers	Accession number	Sequence
*Gfap*	NM_001131020.1	F 5′- ACAGACTTTCTCCAACCTCCAG-3’ R 5′- CCTTCTGACACGGATTTGGT-3’
*S100b*	NM_009115.3	F 5′- CTGGAGAAGGCCATGGTTGC-3’ R 5′- CTCCAGGAAGTGAGAGAGCT-3’
*Aldh1l1*	NM_027406.2	F 5′- CAGTTCTTCAAGGGGTCTGC-3’ R 5′- CAGAATTCGCATCCAAGAGC-3’
*Glul*	NM_008131.4	F 5′- CCGCTCGCTAAGCTGTTACT-3’ R 5′- CTTTGGTGTTAGAGAGGACAACTTT-3’
*Itgam*	NM_001082960.1	F 5′- CAGCCTTTGACCTTATGTCATGG-3’ R 5′- CCTGTGCTGTAGTCGCACT-3’
*Fox3*	NM_001039167.1	F 5′-ACCTACAGCATCGGAACCAT- 3’ R 5′-TTGCTAGTAGGGGGTGAAGC-3’
*Mbp*	NM_001025251.2	F 5′- CTCAGAGGACAGTGATGTGTTT-3’ R 5′- CGCCTTGCCAGTTATTCTTTG-3’
*S100a4*	NM_011311.2	F 5′- GACTCCTCAGATGAAGTGTTGG-3’ R 5′- GTGCGAAGAAGCCAGAGTAA-3’
*Vim*	NM_011701.4	F 5′- GTACCGGAGACAGGTGCAGT-3’ R 5′- TTCTCTTCCATCTCACGCATC-3’
*Lcn2*	NM_008491.1	F 5′- CCCCATCTCTGCTCACTGTC–3’ R 5′- TTTTTCTGGACCGCATTG-3’
*Serpina3n*	NM_009252.2	F 5′- GCAACACCCTGGAAGAGATT-3’ R 5′- CTGGGTCTGTTTCCTCACATAG-3’
*Steap4*	NM_054098.3	F 5′- CCCGAATCGTGTCTTTCCTATAA-3’ R 5′- CCTCGATAGAGCTGCAGAATG-3’
*Hspb1*	NM_013560.2	F 5′- ACAGTGAAGACCAAGGAAGG-3’ R 5′- CTGGAGGGAGCGTGTATTT-3’
*Cxcl10*	NM_021274.2	F 5′- AGTAACTGCCGAAGCAAGAA-3’ R 5′- GCACCTCCACATAGCTTACA-3’
*Timp1*	NM_001044384.1	F 5′- CCTGGGTTTTCATTGGAGGGTTG-3’ R 5′- GTGGCAGAACTTGAGGAGGTC-3’
*Serping1*	NM_009776.3	F 5′- TGATGGCGCCTTTCTTCTAC-3’ R 5′- CCACCTTGGCCTTCAAAGTA-3’
*H2t23*	NM_010398.3	F 5′- GATACCTACGGCTGGGAAATG-3’ R 5′- GTGATGTCAGCAGGGTAGAAG-3’
*Ggta1*	NM_001308300.1	F 5′- TCATCGGGTCCTACCTACAA-3’ R 5′- CTTCAGTCACCTGCTCCATAC-3’
*H2D1*	NM_010380.3	F 5′- CGATGCCTTTGGAGGAGTAT-3’ R 5′- GCATAAGGACGGCAGAATAGA-3’
*S100a10*	NM_009112.2	F 5′- GGGCTTCCAGAGCTTTCTATC-3’ R 5′- CTCCAGTTGGCCTACTTCTTC-3’
*Tgm1*	NM_001161715.1	F 5′- GCCCTTGAGCTCCTCATTG-3’ R 5′- CCCTTACCCACTGGGATGAT-3’
*Il66*	NM_031168.2	F 5′- TGCTGCCACCTTATTTACTCC-3’ R 5′- AGTGGGGCATGATGACAGTT-3’
*Il12b*	NM_001303244.1	F 5′- GCTTCTTCATCAGGGACATCA-3’ R 5′- CTTGAGGGAGAAGTAGGAATG-3’
*Il33*	NM_001164724.2	F 5′- TCCACGGGATTCTAGGAAGA-3’ R 5′- GAGGCAGGAGACTGTGTTAAA-3’
*Ccl2*	NM_011333.3	F 5′- TGAGGCTCAGCACAGCAA-3’ R 5′- ATGGGCTTCCAGAATACCG-3’
*Ccl4*	NM_013652.2	F 5′- GCCCTCTCTCTCCTCTTGCT-3’ R 5′- GGAGGGTCAGAGCCCATT-3’
*Ccl5*	NM_013653.3	F 5′- TGCAGAGGACTCTGAGACAGC-3’ R 5′- GAGTGGTGTCCGAGCCATA-3’
*Thbs1*	NM_011580.4	F 5′- CCCCAACCTTCCCAACTC- 3’ R 5′- GGGTTGTAATGGAATGGACAG-3’
*Thbs4*	NM_011582.3	F 5′- ATGGGACGGCTGAACAAA-3’ R 5′- GCCATCTCCTCTCTGCAAAT-3’
*Sparcl1*	NM_010097.4	F 5′- AAACCATCCCAGTGACAAGG-3’ F 5′- GTCCACCTCGAAGCTGTAGG-3’
*Gpc4*	NM_008150.2	F 5′- CCTTGATTTTGAGTGGAACAATTT-3’ R 5′- AATGTTGAAAGGACCCTCCAG-3’
*Gpc6*	NM_001079844.2	F 5′- CCTTGATTTTGAGTGGAACAATTT-3’ R 5′- AATGTTGAAAGGACCCTCCAG- 3’
*Sparc*	NM_009242.5	F 5′- CACCCAGACTCTGTGCTTATT-3’ R 5′- TCAGGTCTCACAGCATCTTTAC-3’
*Vegfa*	NM_001287056.1	F 5′- CCGCTCGCTAAGCTGTTACT-3’ R 5′- CTTTGGTGTTAGAGAGGACAACTTT-3’
*Bdnf*	NM_007540.4	F 5′- GCATCTGTTGGGGAGACAAG-3’ R 5′- TCACCTGGTGGAACATTGTG-3’
*Ntf3*	NM_001164034.1	F 5′- GGTAATGGAGGGCTTTCTTCTC-3’ R 5′- GCACCGAAGAGAATCAGGTTTA-3’
*Syp*	NM_009305.2	F 5′- CAAGGCTACGGCCAACAG-3’ R 5′- GTCTTCGTGGGCTTCACTG-3’
*Syn2*	NM_001111015.1	F 5′- CCAATCACCGAGAGATGCTTAC-3’ R 5′- CAATGTCCTGGAAGTCGTAGTG-3’
*Dlg4*	NM_007864.3	F 5′- CGCTACCAAGATGAAGACACG-3’ R 5′- CAATCACAGGGGGAGAATTG-3’
*Thbs2*	NM_011581.3	F 5′- GAACCAACCCTTCGGTGTT-3’ R 5′- TGGATTCTCTGGCTCACACA-3’
*Gria1*	NM_001113325.2	F 5′- AGGGATCGACATCCAGAGAG-3’ R 5′- TGCACATTTCCTGTCAAACC-3’
*Gria4*	NM_019691.4	F 5′- CTGCCAACAGTTTTGCTGTG-3’ R 5′- AAATGGCAAACACCCCTCTA-3’
*Tmem119*	NM_146162.3	F 5′- CCACAGCATTGAGGAGTTTG-3’ R 5′- ACAGCTCATCATTTGGCTCA-3’
*CD68*	NM_001291058.1	F 5′- CTGCCAGTCCGAAAATGGAAC-3’ R 5′- CTTCATCCACCGGGGCTATC-3’
*Tnfa*	NM_013693.3	F 5′- CTGTAGCCCACGTCGTAGC-3’ R 5′- TTGAGATCCATGCCGTTG-3’
*Il1β*	NM_008361.4	F 5′- AGTTGACGGACCCCAAAAG-3’ R 5′- AGCTGGATGCTCTCATCAGG-3’
*Il1α*	NM_010554.4	F 5′- CTCTGAGAACCTCTGAAACGTC-3’ R 5′- GAAACTCAGCCGTCTCTTCTT-3’
*C1qa*	NM_007572.2	F 5′- GAGTCCATACCAGAACCACAC-3’ R 5′- ACAGACAAAGGTCCCACTTG-3’
*P2ry12*	NM_027571.4	F 5′- AACACCACCTCAGCCAATAC-3’ R 5′- ACAGCAATGGGAAGAGAACC-3’
*Trem2*	NM_031254.3	F 5′- TGGGACCTCTCCACCAGTT-3’ R 5′- GTGGTGTTGAGGGCTTGG-3’
*Tlr2*	NM_011905.3	F 5′- CACTATCCGGAGGTTGCATATC-3’ R 5′- GGAAGACCTTGCTGTTCTCTAC-3’
*Aif1*	NM_001361501.1	F 5′- GACGTTCAGCTACTCTGACTTT-3’ R 5′- GTTGGCCTCTTGTGTTCTTTG-3’
*Cx3cr1*	NM_009987.4	F 5′- GAGAGATGGCTCAGTGGTTAAG-3’ R 5′- CACAGGAACAGGGAGCTATTT-3’
*Ccl9*	NM_011338.2	F 5′- CTTGAGCTACAGCCATCCTAAC-3’ R 5′- CAGACCACTCTCACAGTTTATCC-3’
*Ccl12*	NM_011331.3	F 5′- GTTCCTGACTCCTCTAGCTTTC-3’ R 5′- GCATCTGGTCCAGCCAATA-3’
*Ccl6*	NM_009139.3	F 5′- GGGACAGCATTCTGAACTCTAC-3’ R 5′- CTCACTCCTCCCTGATTCTCT-3’
*Il10*	NM_010548.2	F 5′- AGAAAAGAGAGCTCCATCATGC-3’ R 5′- CGGAATTTCTGGGATTCAGCTTC-3’
*Gapdh*	NM_001289726.1	F 5′- AACAGCAACTCCCACTCTTC-3’ R 5′- CCTGTTGCTGTAGCCGTATT-3’

### Protein extraction and Western blotting

The 70–80% confluent primary cultures were washed with ice-cold PBS, lysed with cell lytic MT Mammalian cell lysis buffer containing a protease inhibitor cocktail and kept on ice for 10 min. Cells were scrapped, collected in micro centrifuge tubes and centrifuged at 4 °C, 15,000 rpm for 30 min. The supernatant was collected in fresh micro centrifuge tube. Protein concentration was determined through BCA assay. For Western blots, protein samples were prepared with 1X SDS sample loading buffer, and denatured at 85 °C for 15 min. Equal amounts of protein (40 µg) were then loaded onto 10–12% tris–glycine polyacrylamide gel and run in 1X running buffer at 100 V. After completion of electrophoresis, the transfer onto the PVDF membrane (activated in methanol) was conducted at 16 V for 60 min, then membranes were washed with TBST (10 mM Tris, pH 8.0, 150 mM NaCl and 0.01% Tween 20) and blocked with 5% non-fat milk for 1 h. Membranes were washed thrice with TBST, probed overnight with GFAP (1:3,000), LCN2 (1:2,000), Iba1 (1:2,000), Synaptophysin (1:3,000), PSD-95 (1:2,000) and β-actin (1:10,000) antibodies, then washed four times with TBST and incubated with rabbit or mouse HRP-conjugated secondary antibodies. Protein bands were visualized using the chemiluminescent Imager (Thermo-Scientific) with a Supersignal West Femto Maximum Sensitivity Substrate. Densitometry analysis was performed using Bio-Rad Quantity One image analysis software (Bio-Rad, Hercules, CA).

### Immunocytochemistry

Cells cultured in chamber slides/coverslips were fixed in 4% paraformaldehyde for 30 min at RT, washed with PBS followed by permeabilization in methanol for 30 min, then washed, blocked with blocking serum (3% BSA + 1% NGS in PBS) for 2 h at RT and incubated at 4 °C with primary antibodies at the following dilutions: GFAP (1:1000), s100β (1:1000), Iba1 (1:1000), MBP (1:1000), NeuN (1:200), LCN2 (1:200), Olig2 (1:1000), Cd11b (1:1000), MAP2 (1:1000), SYP (1:500 or 1:200 for co-cultures), PSD95 (1:500), and Drebrin (1:1000). After four washes in PBST (PBS + 0.1% Tween-20), cells were incubated with a cocktail of the secondary antibodies (Alexa Fluor 488 goat anti-rabbit, Alexa Fluor 488 goat anti-chicken, Alexa Fluor 488 goat anti-mouse, Alexa Fluor 546 goat anti-rabbit, Alexa Fluor 546 goat anti-mouse, Alexa Fluor 647 goat anti-mouse IgG conjugate, all at 1:400 dilution) for 2 h. Chamber slides/coverslips were then washed four times in PBST and mounted in VECTASHIELD medium with DAPI (Vector Laboratories, Burlingame, CA). Cells were imaged using a Nikon Eclipse TE2000-U inverted microscope (Nikon Instech Co. Ltd., Kawasaki, Kanagawa, Japan) or Leica confocal microscope SP8 (Leica Microsystems Inc., Buffalo Grove, IL), and the NIS-Elements microscope imaging software (Nikon Instech Co.) or Leica LAS X software (Leica Microsystems Inc.).

### Analysis of astrocyte morphology

For identifying the area, perimeter and number of processes, non-overlapping astrocytes immunostained for GFAP were imaged using a Nikon inverted microscope (20X objective) and analysed with ImageJ software (National Institutes of Health, Bethesda, MD). Equal numbers of cells (30,000) were seeded per well of a chamber slide and the entire surface of the chamber slide was examined for image capturing. Images of 5 fields of view were selected per well of each chamber slide; 15 to 20 images per chamber slide per one experimental condition were taken. After subtraction of the background and threshold adjusting, non-overlapping cells from a 20X image (5–7 cells per field of view) were selected. The expression levels of LCN2 were assessed by determining integrated density using the RGB plugin of the Image-J software. Three independent experiments that employed astrocyte cultures originating from individual animals were performed.

### Analysis of neuronal morphology

The dendritic length, dendritic branch and dendritic number of the primary dendrites evolving from the soma and dendritic branches were analysed using the Neurite Quant plugin in ImageJ. Images of MAP2-positive cells were captured using a Nikon inverted microscope and 20X objective. In primary neuronal culture, images of neurons with soma in 5 random fields of view per well of 4 well-chamber slides (15–20 images per chamber slide) were analysed. In co-cultures, images of 10 random fields per coverslip with 5–7 cells per field of view were analysed per experimental group. Three independent experiments were performed.

### Synaptic puncta quantification

Synapse quantification was calculated using custom-written plugin, Puncta Analyzer as described before [59]. Briefly, cells in primary and co-cultured neurons were fixed in 4% PFA and co-immunostained for synaptophysin (SYP), PSD-95 and MAP2. Confocal images were taken using a Leica confocal microscope SP8 (Leica Microsystems Inc.), 63X oil-immersion objective lens at 1024 × 1024 pixel resolution 2X zoom and zoom factor 1.5 corresponding to a voxel dimension 0.13 µm × 0.13 µm × 0.32 µm in X, Y, and Z plans. Cell bodies were centred in the field of view and Z-stack dimensions were set manually by tracking MAP2-positive neurons including dendrites and soma. Synapses were quantified by analysing co-localization of SYP and PSD-95 puncta using a custom based plug-in Puncta Analyzer in ImageJ. Background was removed separately from the red and green channel using the rolling ball background subtraction algorithm, then thresholding was performed for detecting discrete puncta, and puncta were identified in red and green channels using the Puncta Analyzer plugin. Co-localized puncta were counted as a synapse. Three independent experiments were performed. For each independent experiment, 15 to 20 neurons from at least two coverslips (or one 4-well chamber slide) were analysed per experimental conditions. For each independent culture, similar regions of interest (all dendrites of neurons including soma) were assessed for co-localization and multiple dendritic fragments were sampled within a particular neuron.

### Dendritic spine analysis

Cells in primary and co-cultured neurons were fixed in 4% PFA and co-immunostained for Drebrin and MAP2. Confocal images were taken using Leica confocal microscope SP8, 63X oil-immersion objective lens at 2X zoom and 1024 × 1024 pixel resolution with the Z stack acquisition. Images were analyzed using Imaris software (Bitplane, Zurich, Switzerland). Dendritic spine densities were calculated by quantifying the number of spines per 10 μm length of dendritic segments. Three independent experiments were performed and 30 dendritic segments (10 μm in length) in each condition were analyzed.

### Analysis of gene expression by nanostring

After euthanasia by CO2 asphyxiation, brains were immediately extracted and kept ice-cold during dissection. Brains were sliced using rodent brain slicer matrix (Zivic Instruments, Pittsburg, PA). Cortex samples were collected from 2 mm central coronal sections of each brain. RNA isolation from cortex, cultured and acutely isolated astrocytes was performed as described before [50]. RNA samples were processed by the Institute for Genome Science at the University of Maryland School of Medicine using the nCounter custom-designed Nanostring gene panel (Nanostring Technologies, Seattle, WA), which consisted of genes that are expressed predominantly by astrocytes (www.brainrnaseq.org). Only samples with an RNA integrity number RIN > 7.2 were used for Nanostring analysis. All data passed quality control, with no imaging, binding, positive control, or CodeSet content normalization flags. The analysis of data was performed using nSolver Analysis Software 4.0. Ten house-keeping genes (*Xpnpep1, Lars, Tbp, Mto1, Csnk2a2, CCdc127, Fam104a, Aars, Tada2b, Cnot10*) were used for normalization of gene expression.

### Statistics

Statistical analyses was performed with GraphPad PRISM 7 software (GraphPad software, Inc., San Diego, CA) and is described in Table 2.

**Table 2:** Statistical analysis

Figure	Type of statistical analysis
S1C	Unpaired *t* test, two tailed, type 2, n = 3
1A	Unpaired *t* test, two tailed, type 2, n = 3
1B	Unpaired *t* test with Welch's correction, two-tailed, t = 5.899, df = 2, n = 3
1C	Unpaired *t* test with Welch's correction, two tailed, t = 4.46, df = 3.921 for area, t = 3.692, df = 2.541 for perimeter, t = 3.474, df = 4 for processes number, n = 3
1D	Unpaired *t* test, two tailed, type 2, n = 3
1E	Unpaired *t* test with Welch's correction, two tailed, t = 12.36, df = 2, n = 3
1F	Unpaired *t* test with Welch's correction, two tailed, t = 5.535, df = 2, n = 3
1G	Unpaired *t* test, two tailed, type 2, n = 3
1H	Unpaired *t* test with Welch's correction, two tailed, t = 7.06, df = 3.29, n = 3
2A	Unpaired *t* test with Welch's correction, two tailed, t = 8.798, df = 2, n = 3
2B	Unpaired *t* test with Welch's correction, two tailed, t = 6.376, df = 2, n = 3
3C	Unpaired *t* test with Welch's correction, two tailed, t = 8.311, df = 2.461 for spine size and t = 3.744, df = 2.50 for spine density, n = 3
3A	Unpaired *t* test, two tailed, type 2, n = 3
3D	Unpaired *t* test with Welch's correction, two tailed, t = 4.68, df = 3.10 for area of soma, t = 6.34, df = 2.909 for dendrite length and t = 7.702, df = 3.764 for dendritic branch number, n = 3
3E	Unpaired *t* test, two tailed, type 2, n = 3
3F	Unpaired *t* test, two tailed, type 2, n = 3
4B	Unpaired *t* test with Welch's correction, two tailed, t = 5.599, df = 2, n = 3
4C	Unpaired *t* test with Welch's correction, two tailed, t = 5.939, df = 2 for SYP and t = 4.618, df = 2 for PSD95, n = 3
4D	Unpaired *t* test with Welch's correction, two tailed, t = 3.142, df = 3.978 for spine size and t = 3.888, df = 2.50 for spine density, n = 3
S3C	Unpaired *t* test, two tailed, type 2, n = 3
S3D	Unpaired *t* test, two tailed, type 2, n = 3
S3E	Unpaired *t* test with Welch's correction, two tailed, t = 3.142, df = 3.978, n = 3
S3F	Unpaired *t* test with Welch's correction, two tailed, t = 12.92, df = 2.9 for TNFα, t = 10.3, df = 2.074 for IL-1α and t = 5.08, df = 2.403 for C1q, n = 3
5A	Unpaired *t* test with Welch's correction, two tailed, t = 6.79, df = 3,449 for area, t = 7.052, df = 3.41 for perimeter and t = 3.889, df = 2.56 for processes number of astrocytes, n = 3
5B	Unpaired *t* test, two tailed, type 2, n = 3
5C	Unpaired *t* test, two tailed, type 2, n = 3

## Results

### Isolation and purification of primary astrocytes from adult mouse brains

To examine the pathophysiological role of astrocytes in prion diseases, first we optimized a protocol for isolation and culturing of primary astrocytes from adult mouse brains (220–300 day old C57Bl/6J mice). The protocol involved cell dissociation, removal of debris and myelin, followed by enrichment of cultured cells with astrocytes (Additional file 1: Figure S1A). The purity of the primary cultures was confirmed by co-immunostaining for the astrocyte-specific marker GFAP and the markers of microglia, oligodendrocyte and neurons (Iba1, MBP and NeuN, respectively). More than 95% of cells were found GFAP-positive and Iba1-, MBP- and NeuN-negative (Additional file 1: Figure S1B). In addition, co-immunostaining for GFAP and another astrocyte-specific marker, S100β, revealed that in primary cultures, the vast majority of cells were positive for both markers (Additional file 1: Figure S1B). To validate cell composition further, the expression of astrocyte- (*Gfap, S100b, Aldh1l1* and *Glul)*, microglia- (*Itgam*), neuron- (*Fox3*), oligodendrocyte- (*Mbp*) and fibroblast-specific (*S100a4*) genes were analyzed using RT-qPCR. When normalized relative to the mouse cortex tissue used as a reference, the astrocyte primary cultures were enriched with the transcripts of astrocyte specific genes, whereas the expression of markers of other cell types was found to be considerably low (Additional file 1: Figure S1C).

For examining the extent to which transcriptome of primary astrocytes changes as a result of culturing in vitro, transcriptomes of cultured and acutely isolated astrocytes were analyzed using custom-designed Nanostring panel composed of astrocyte-specific genes. As expected, both primary astrocyte cultures and acutely isolated astrocytes were enriched with transcripts of astrocyte-specific genes, when compared to the bulk brain cortex tissues or primary microglia cultures (Additional file 2: Figure S2). Clustering analysis revealed that primary and acutely isolated astrocytes displayed very similar patterns of gene expression. Indeed, astrocytes prepared via these two procedures did not segregate into distinct subclusters but formed one cluster (Additional file 2: Figure S2). Perturbation in expression of genes responsible for astrocyte physiological functions resulting from in vitro culturing could not be excluded. Nevertheless, these results indicate that, under the culturing conditions employed in the current work, primary astrocytes recapitulate the transcriptome profile of acutely isolated astrocytes fairly well.

### Astrocytes isolated from prion-infected mice exhibit a reactive phenotype

As a source of primary astrocytes from animals infected with prions, we used clinically sick C57Bl/6J mice (176–238 days post-inoculation, 220–283 day old) infected intraperitoneally with 22L mouse-adapted prion strain. While mice challenged intraperitoneally develop disease slower in comparison to the intracranially-challenged mice, the intraperitoneal route is more physiological and does not cause neuroinflammation due to direct brain injuries associated with the intracranial injections.

**Fig. 1:**
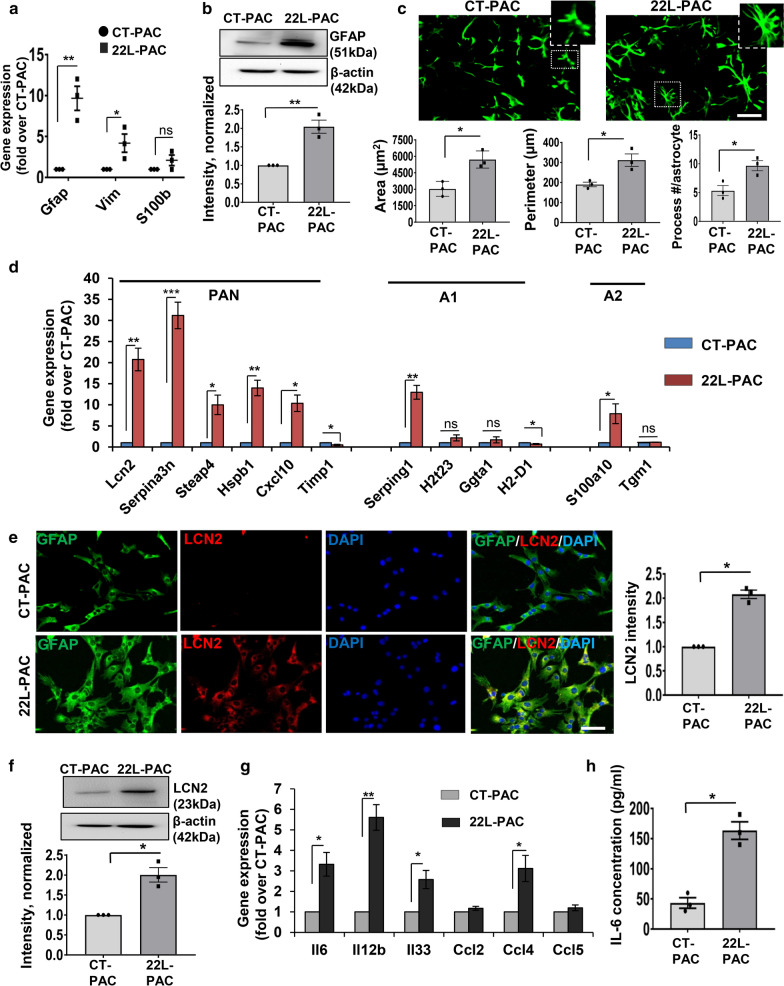
Astrocytes isolated from 22L-infected mice exhibit a reactive phenotype. **a** Analysis of gene expression in 22L-PACs, normalized by the expression of the same genes in CT-PACs, using qRT-PCR. **b** Representative Western blots and densitometric analysis of GFAP expression normalized per expression of β-actin in CT-PACs and 22L-PACs. **c** Representative images of CT-PACs and 22L-PACs stained for GFAP, and morphometric analyses of cell area, perimeter and process number in astrocytes from CT-PACs and 22L-PACs. Insets show magnified images. **d** Analysis of expression of PAN-, A1- and A2-specific genes in 22L-PACs normalized by the expression levels in CT-PACs using qRT-PCR. **e** Representative images of co-immunostaining of CT-PACs and 22L-PACs for GFAP (green), LCN2 (red) and nuclei (DAPI, blue), and quantification of integrated fluorescence intensity of LCN2 in CT-PACs and 22L-PACs. **f** Representative Western blots and densitometric analysis of LCN2 expression normalized per expression of β-actin in CT-PACs and 22L-PACs. **g** Analysis of expression of pro-inflammatory genes in 22L-PACs normalized by the expression levels in CT-PACs using qRT-PCR. **h** Analysis of IL-6 concentration in media conditioned by CT-PACs and 22L-PACs. In panels **a**, **d** and **g**, *Gapdh* was used as a housekeeping gene. In panels **a**–**h**, data represent means ± SE, n = 3 independent cultures isolated from individual animals, ***p < 0.001, **p < 0.01, *p < 0.05, and ‘ns’ non-significant (two tailed, unpaired *t* test). Scale bar = 50 µm for panels **c** and **e**

Primary astrocyte cultures derived from 22L animals (22L-PAC) showed elevated levels of GFAP expression at mRNA and protein levels relative to the primary astrocyte cultures derived from age-matched control animals (CT-PAC) (Fig. 1a,b). GFAP is a traditional marker of reactive astrocytes that has been employed for assessing reactive astrogliosis associated with prion diseases [60–63]. Consistent with upregulation of GFAP expression, 22L-derived astrocytes had enlarged, hypertrophic morphology characterized by enlarged cell area, cell perimeter and an increase in the number of processes (Fig. 1c). Among two additional astrocytic markers tested, vimentin (*Vim*) mRNA showed statistically significant upregulation in 22L cultures relative to the control astrocytes, whereas upregulation of S100b was modest and not statistically significant (Fig. 1a). These results are consistent with the previous results, documenting significant upregulation of *GFAP* and *Vim* across brain regions in prion-infected animals and modest, region-dependent upregulation of S100b [47].

Previous studies revealed that in prion diseases, astrocytes do not exhibit uniform polarization into A1- or A2-states, but display mixed phenotypes characterized by the upregulation of most PAN-specific markers, as well as several A1- and A2-specific markers [21, 50, 64]. In agreement with these results, astrocytes isolated from 22L-infected mice showed statistically significant upregulation of PAN-reactive markers *Lcn2, Serpina3n, Steap4, Cxcl10, Hspb1, Vim*, the A1 marker *Serping1* and the A2 marker *S100a10* (Fig. 1a,d). The upregulation of LCN2 expression on a protein level was verified using Western blot and immunocytochemistry (Fig. 1e,f). A few markers (A1: *H2t23, Ggta1, H2D1;* A2*: Tgm1;* PAN: *Timp1*) that were found to be upregulated in prion infected animals [50] showed similar or lower expression levels in 22L-PACs relative to the CT-PACs (Fig. 1d). Apparent discrepancies between primary cultures and brains have to be considered with caution, because in a brain, multiple cell types contribute to the expression of the A1/A2/PAN-specific markers including *Ggta1, Tgm1* and *Timp1*.

To assess the neuroinflammation status of 22L-PACs, expression of several interleukins and cytokines known to be upregulated in prion-infected animals was examined [34, 50]. In comparison to CT-PACs, 22L-PACs expressed higher levels of *Il6, Il12b, Il33* and *Ccl4*, but not *Ccl2* or *Ccl5* (Fig. 1g). To verify results of gene expression, IL-6 was tested in conditioned media and found to be elevated in the media conditioned by 22L astrocytes relative to the media from control cultures (Fig. 1h). While the classical pathway activated by IL-6 is considered anti-inflammatory, increased concentrations of IL-6 were shown to trigger an alternative, pro-inflammatory pathway that was linked to neurodegeneration [65]. In summary, astrocytes isolated from 22L-infected animals preserved, at least in part, reactive phenotypes associated with prion diseases characterized by hypertrophic morphology and upregulation of *Gfap*, *Vim*, the majority of PAN-specific markers as well as proinflammatory interleukins and cytokines.

### 22L-derived astrocytes impair neuronal growth and synapse maturation

Under physiological conditions, astrocytes perform a variety of physiological functions that are essential for maintaining healthy, functional neurons [52–54]. As astrocytes are critical to neuronal function, co-culturing of cortical primary neurons with astrocytes in vitro is known to provide positive effects on neuronal growth and synapse maturation [66]. Therefore, to test whether reactive astrogliosis associated with prion disease undermines the supportive role of astrocytes, first we examined the effect of reactive astrocytes on neurons using neuron-astrocyte co-cultures. Primary cortical neurons were isolated from P1–P2 pups and plated on 50% confluent CT- or 22L-PACs.

**Fig. 2:**
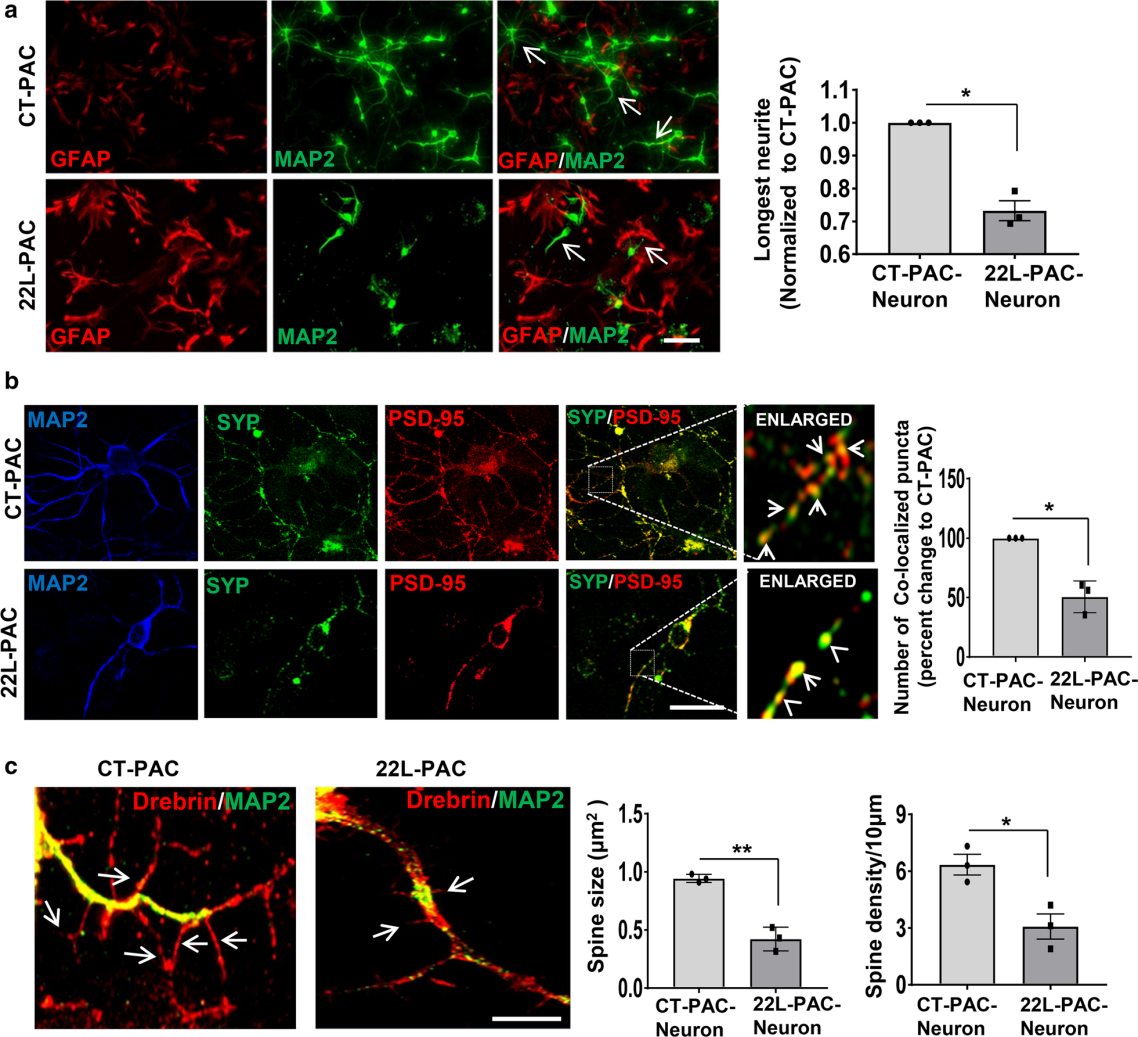
22L-derived reactive astrocytes show impairment in supporting neuronal growth. Primary cortical neurons were plated on CT-PACs or 22L-PACs and co-cultured for 10–12 days*.* **a** Representative fluorescent images of neuron-astrocyte co-cultures co-immunostained for MAP2 (green) and GFAP (red), and quantification of neurite length. **b** Representative fluorescent images of cortical neuronal cells co-cultured with CT- PACs or 22L-PACs, and co-immunostained for pre- and post-synaptic markers synaptophysin (SYP, green) and PSD95 (red), respectively, and MAP2 (blue). Co-localization between SYP and PSD95 was analyzed for quantification of synapses in neuronal cells co-cultured with CT-PACs or 22L-PACs (yellow puncta). **c** Representative images of cortical neuronal cells co-cultured with CT-PACs or 22L-PACs, and co-immunostained with a spine marker Drebrin (red) and MAP2 (green). Quantification of spine size and density in neurons co-cultured with CT-PACs and 22L-PACs. In **a**–**c**, data represent mean ± SE, n = 3 independent experiments in which CT-PACs or 22L-PACs were isolated individual animals, 50 neurons (in **a** and **b**) and 40 neurons (in **c**) per conditions were analyzed, **p < 0.01 and *p < 0.05 (two tailed, unpaired *t* test with Welch's correction). Scale bars = 50 µm in **a**, 25 µm in **b**, and 10 µm in **c**

The morphology of neurons co-cultured with 22L-PACs was markedly different in comparison to that of neuronal cells cultured with CT-PACs. In particular, neurons co-cultured with 22L-PACs were characterized by reduced number of neurites and significantly shorter neurites (Fig. 2a). Moreover, reduction in the colocalization of pre- and postsynaptic proteins, synaptophysin and PSD95, respectively, was observed in neurons grown with 22L-PACs (Fig. 2b). In addition, spine density and size were also declined in neurons grown with 22L-PACs (Fig. 2c). In summary, 22L-derived reactive astrocytes had a negative impact on neurite outgrowth, synapse formation and spine density in primary neurons.

### Factors released by reactive astrocytes impairs synapse integrity and spine density

**Fig. 3:**
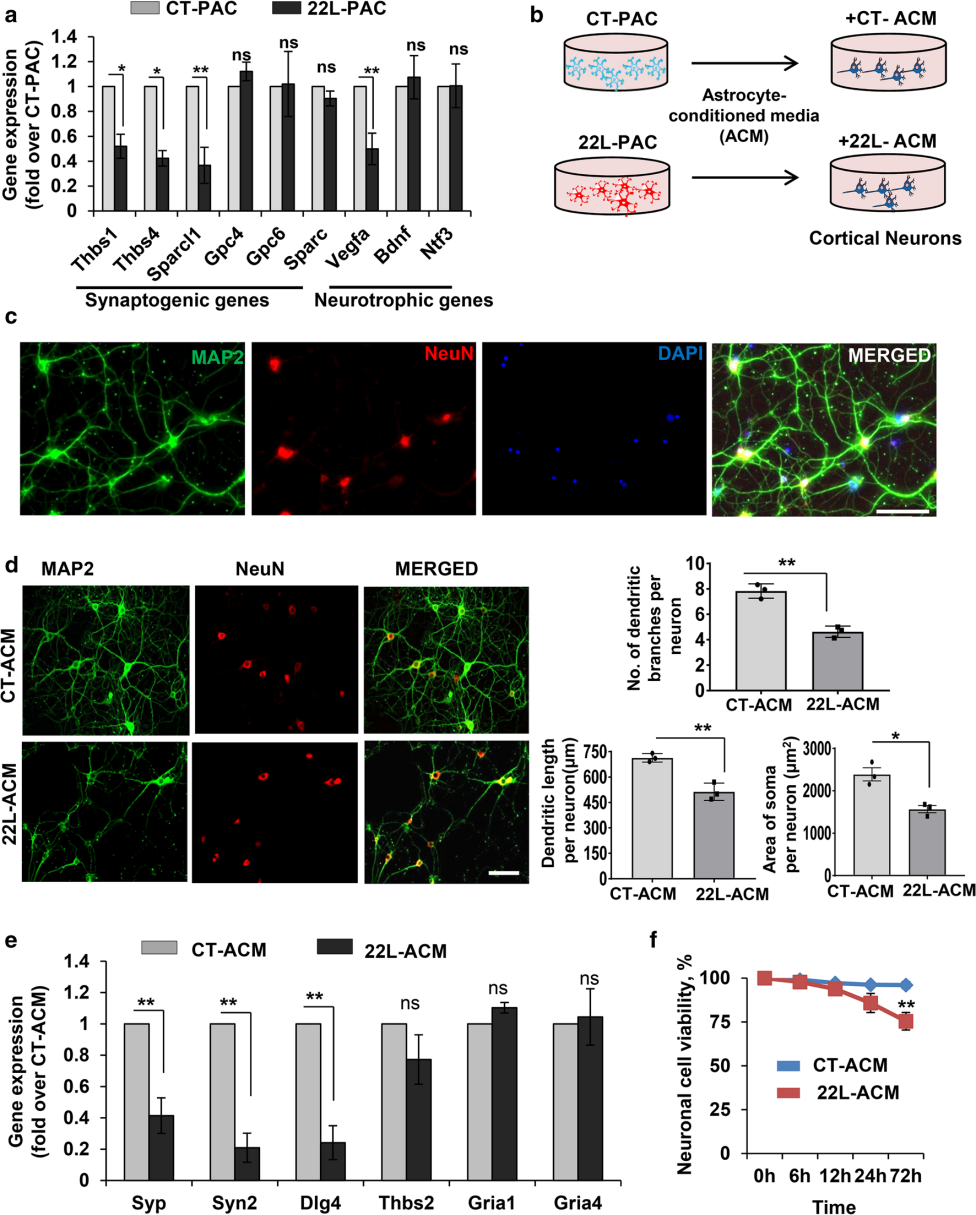
Deleterious effects of factors released by 22L-astrocytes on neuronal morphology, viability and expression of functional genes. **a** Analysis of expression of synaptogenic and neurotrophic genes in 22L-PACs normalized by the expression levels in CT-PACs using qRT-PCR. **b** Schematic illustration of experiments on treatment of mouse primary cortical neuronal cultures with astrocyte-conditioned media (ACM) collected from CT-PACs and 22L-PACs. **c** Representative images of primary neuronal cultures isolated from P1–P2 mice and co-immunostained for MAP2 (green), NeuN (red) and DAPI (blue). **d** Left: representative images of primary neuronal cultures treated with CT-ACM and 22L-ACM for 72 h and co-immunostained for MAP2 (green) and NeuN (red). Right: quantification of neuronal morphology using MAP2 fluorescence in primary neuronal cultures treated with CT-ACM and 22L-ACM for 72 h. **e** Analysis of expression of *Syp, Syn2, Dlg4, Thbs2, Gria1* and *Gria4* in primary neuronal cultures treated with 22L-ACM for 72 h and normalized by the expression levels in cultures treated with CT-ACM using qRT-PCR. **f** Cell viability in primary neuronal cultures assessed by MTT assay as a function of incubation time with CT-ACM or 22L-ACM. In panels **a** and **e**, *Gapdh* was used as housekeeping gene. In panels. **a**, **d**–**f**, data represent means ± SE, n = 3 independent astrocyte or microglia cultures isolated from individual animals, ***p < 0.001, **p < 0.01, *p < 0.05, and ‘ns’ non-significant (two tailed, unpaired student *t* test). Scale bar = 50 µm

Next, expression of astrocyte-specific genes responsible for formation and maintenance of synapses and genes encoding neurotrophic factors was analyzed in 22L-PACs and CT-PACs. Expression of secreted adhesive glycoproteins that are important for neurogenic and synaptogenic functions (*Thbs1*, thrombospondin-1; *Thbs4*, thrmbospondin-4; *Sparcl1*, hevin) were downregulated in 22L-PACs compared to CT-PACs (Fig. 3a). Expression of glypicans (*Gyp4* and *Gyp6*), cell surface heparin sulfate proteoglycans involved in developmental neurogenesis [67] did not change (Fig. 3a). Among the tested neurotrophic factors with supportive roles [68], vascular endothelial growth factor A (*Vegfa*) was downregulated in 22L-PAC relative to CT-PAC (Fig. 3a), whereas the expression levels of brain-derived neurotrophic factor (*Bdnf*) and neurotrophin-3 (*Ntf3*) did not change in 22L-PACs relative to CT-PACs (Fig. 3a).

The above results suggested that the negative effect of reactive astrocytes on neurons was mediated via secreted factors. Therefore, next we examined whether neuronal morphology, viability and gene expression in primary neuronal cultures was affected by factors released by astrocytes (Fig. 3b,c). Dramatic differences in morphology were observed in primary neurons treated with astrocyte-conditioned media (ACM) from 22L-PACs (22L-ACMs) in comparison to those treated with conditioned media from CT-PACs (CT-ACMs) (Fig. 3d). In particular, cells incubated with 22L-ACMs displayed a significant reduction in dendritic branch number, dendritic length and soma area in comparison to the neuronal cultures treated with CT-ACMs (Fig. 3d). Moreover, significant downregulation of pre-synaptic genes *Syp*, *Syn2* and post-synaptic gene *Dlg4* (Discs Large Homolog 4 or PSD95*)* was also found in 22L-ACM-treated neuronal cultures in comparison to the CT-ACM-treated neurons (Fig. 3e). Finally, cell viability declined in neuronal cultures treated with 22L-ACM (Fig. 3f).

**Fig. 4:**
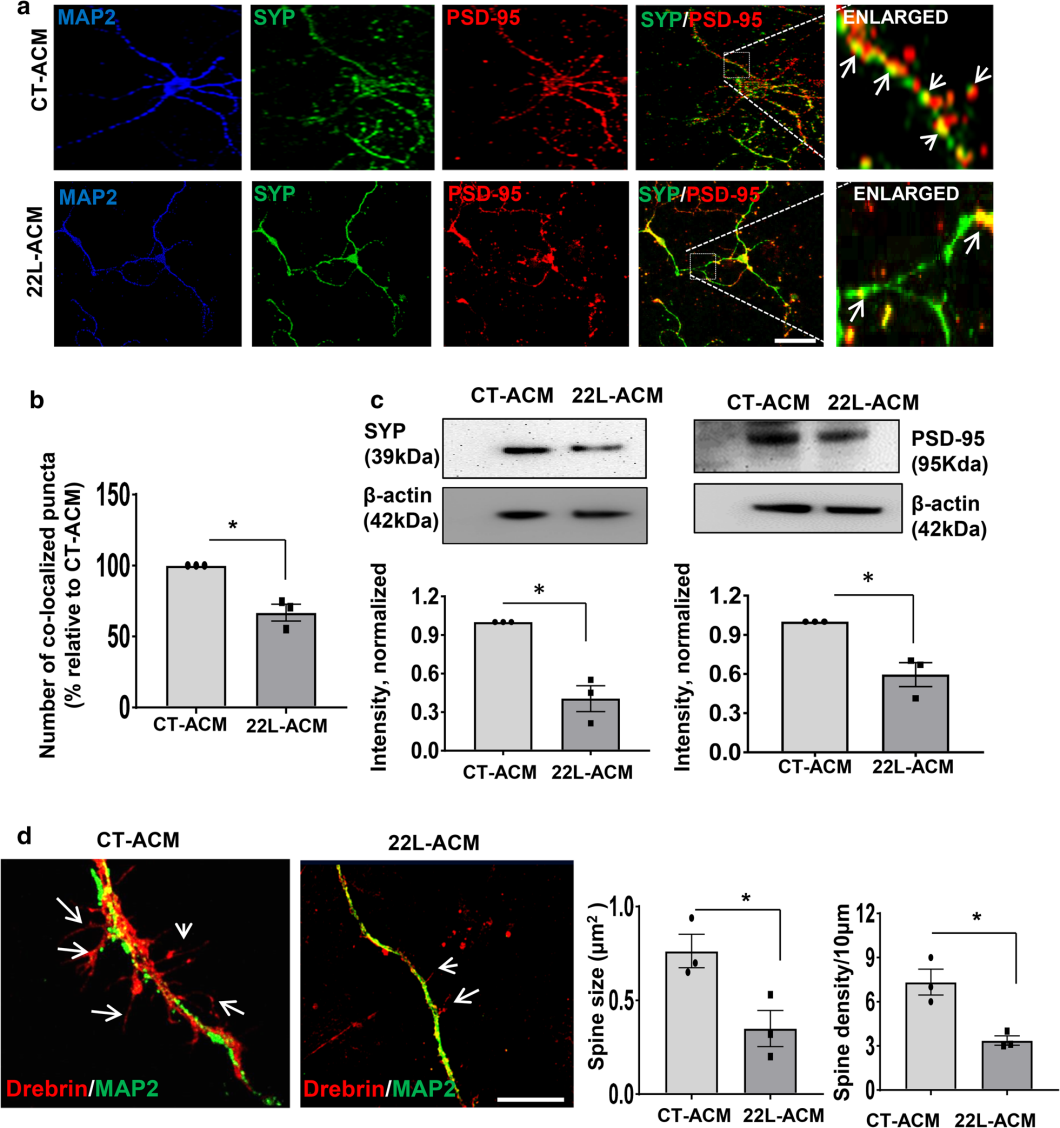
Factors released by 22L-derived reactive astrocytes impairs synapse integrity and spine density. **a** Representative images of primary cortical neurons treated with CT-ACM or 22L-ACM for 72 h and co-immunostained for the pre- and post-synaptic markers synaptophysin (SYP, green) and PSD-95 (red), respectively, and MAP2 (blue). Arrows point at puncta of co-localization of synaptophysin and PSD-95. **b** Quantification of co-localized puncta in CT-ACM- and 22L-ACM-treated primary neurons. **c** Representative Western blots and densitometric analysis of synaptophysin (SYP) and PSD-95 expression normalized per expression of β-actin in neuronal cultures treated with CT-ACM or 22L-ACM. **d** Representative images of neuronal cultures treated with CT-ACM or 22L-ACM for 72 h and co-immunostained for a spine marker Drebrin (red) and MAP2 (green). Arrows point at the secondary dendritic spine branches. Quantification of spine size and density in primary neurons incubated with CT-ACM or 22L-ACM. In **b**–**d**, data represent mean ± SE, n = 3 independent experiments in which CT-ACM or 22L-ACM was collected from cultures established form individual animals, 50 neurons (in **b**) and 40 neurons (in **d**) were counted for each condition. *p < 0.05 (two tailed, unpaired student *t* test with Welch's correction). Scale bar = 25 µm for **a** and 10 µm for **d** panels

Downregulation of pre- and post-synaptic genes raises the question of whether synapse integrity and spine density were also impaired by factors released by reactive astrocytes. Analysis of co-localization of pre- and post-synaptic proteins synaptophysin and PSD95, respectively, using confocal microscopy revealed a significant reduction in the synaptic puncta in primary neurons treated with 22L-ACMs relative to neurons cultured in the CT-ACMs (Fig. 4a,b). Furthermore, Western blot showed that expression levels of synaptophysin and PSD95 was lower in 22L-ACM-treated neurons relative to the CT-ACM-treated neurons (Fig. 4c). For examining spine morphology, co-immunostaining for MAP2 and Drebrin, a spine protein that has been implicated in memory loss in Alzheimer’s disease [69, 70], was analyzed in primary neuronal cultures. Substantial decline in spine size and density was observed in neurons treated with 22L-ACM relative to those incubated with CT-ACM (Fig. 4d). To summarize, the above experiments demonstrated that reactive astrocytes isolated from prion-infected animals had deleterious effects on neurons, characterized by strong synaptotoxic activity. These effects were mediated by astrocyte-secreted factors.

### 22L-derived primary microglia exhibit elevated expression of pro-inflammatory genes and secreted factors

According to the recent hypothesis by Barres and co-workers, reactive states of astrocytes are induced by reactive, proinflammatory microglia [3, 71]. To examine the relationship between reactive microglia and astrocytes, we adapted protocols for isolating microglia from adult C57Bl/6J mice and establishing primary microglia cultures (Additional file 3: Figure S3A). The purity of primary cultures was established using co-immunostaining for the microglial marker CD11b with markers of astrocytes (GFAP), oligodendrocytes (Olig2) or neurons (NeuN). 95% of cells were found to be CD11b-positive and GFAP-, Olig2- and NeuN-negative (Additional file 3: Figure S3B). Moreover, analysis of gene expression using RT-qPCR confirmed that, in comparison to the adult aged-matched brain cortical tissues, primary cultures had an enriched expression of microglia specific genes (*Itgam, Tmem119* and *Cd68)*, while substantially reduced expression of markers of astrocytes (*Gfap*), oligodendrocytes (*Mbp*) and neurons (*Fox3*) (Additional file 3: Figure S3C).

For testing whether microglia preserves a pro-inflammatory phenotype in vitro, we compared the expression of pro-inflammatory genes in primary microglia cultures originating from clinically sick 22L animals (22L-PMC) with that of age-matched controls (CT-PMC). When compared to CT-PMCs, 22L-PMCs showed upregulation in expression of proinflammatory genes (*Tnfa*, *Il1α, Il1β, Il10, Ccl2, Ccl4, Ccl6, Ccl9, Ccl12, Aif1),* the genes involved in innate immune response (*C1qa, Tlr2)* along with *P2ry12,* a microglial homeostatic gene which was found to be upregulated in prion diseases (Additional file 3: Figure S3D) [50]. Western blot confirmed that 22L-PMCs had higher expression of Iba1, a marker of reactive microglia, relative to Iba1 expression in CT-PMCs (Additional file 3: Figure S3E). Moreover, in comparison CT-PMCs, 22L-PMCs secreted considerably higher quantities of pro-inflammatory factors IL-1α, TNF-α and C1q into media (Additional file 3: Figure S3F).

### 22L-derived reactive microglia induce a pro-inflammatory state in primary astrocytes

**Fig. 5:**
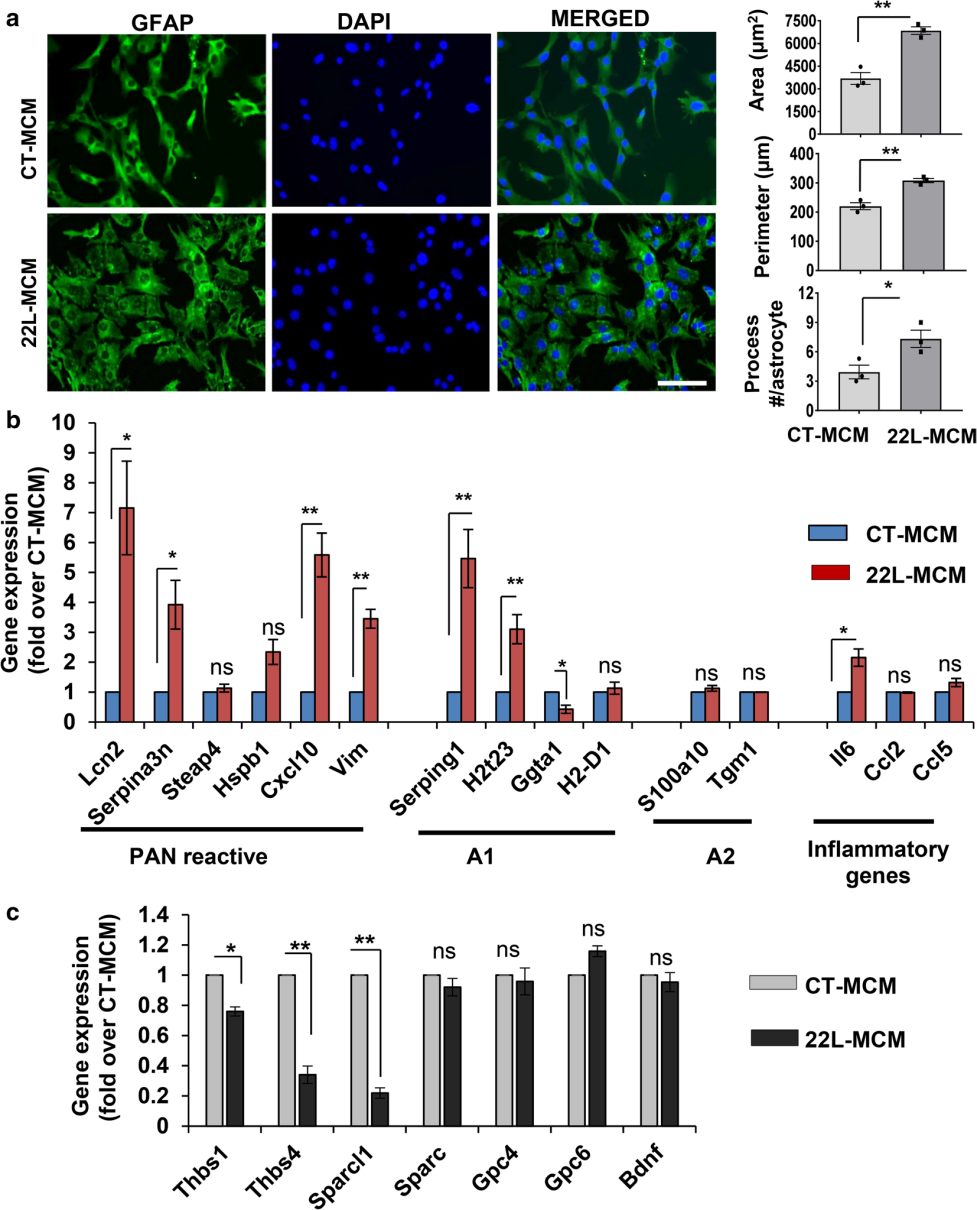
22L-derived reactive microglia induce a pro-inflammatory state in primary astrocytes. **a** Left: representative images of primary astrocyte cultures isolated from adult (200–300 days old) C57 Black mice, treated with CT-MCM or 22L-MCM for 72 h, and co-immunostained for GFAP (green) and nuclei (DAPI, blue). Right: morphometric analysis of astrocytes following treatment of primary cultures with CT-MCM or 22L-MCM and staining for GFAP. Data represent means ± SE, n = 3 independent experiments, **p < 0.01 and *p < 0.05 (two tailed, unpaired student *t* test with Welch's correction). Scale bar = 50 µm. **b** and **c**. Analysis of expression of PAN-, A1- and A2-specific genes (**b**) and synaptogenic genes (**c**) in primary astrocyte cultures following treatment with CT-MCM or 22L-MCM for 72 h using qRT-PCR. *Gapdh* was used as housekeeping gene. Data represent means ± SE, n = 3 independent experiments, i.e. astrocyte cultures isolated from individual animals was treated by CT-ACM or 22L-ACM collected from cultures also established form individual animals, ***p < 0.001, **p < 0.01 and *p < 0.05 and ‘ns’ non-significant (two tailed, unpaired student *t* test)

For testing whether factors secreted by 22L-PMCs dictate astrocyte phenotype, we examined the effect of microglia conditioned medium (MCM) on primary cortical astrocytes isolated from adult C57Bl/6J mice. In contrast to CT-MCMs, 22L-MCMs induced phenotypic changes in primary astrocytes indicative of reactive astrogliosis. In addition to morphological changes characterized by enlarged cell area, cell perimeter and an increase in number of processes (Fig. 5a), 22L-MCM-treated astrocytes also showed elevated levels of PAN-reactive genes (*Lcn2, Serpina3n, Cxcl10, Vim*), A1-specific markers (*Serping1* and *H2t23*) and proinflammatory gene *Il6*, when compared to CT-MCM-treated astrocytes (Fig. 5b). Moreover, astrocytes treated with 22L-MCMs showed down-regulation of the same neurogenic and synaptogenic genes (*Thbs1*, *Thbs4* and *Sparcl1*) that were downregulated in astrocytes purified from 22L-infected animals (Fig. 5c). These results demonstrated that factors secreted by reactive microglia isolated from 22L-infected animals trigger phenotypic changes in normal astrocytes, driving them into reactive states resembling the phenotype of astrocytes in prion-infected brains.

## Discussion

Loss of functional synapses is the early sign of neuronal degeneration and a primary cause of pathological symptoms in prion diseases [72–80]. According to the mechanisms on direct toxicity, binding of PrP^Sc^ to PrP^C^ and/or other molecules on neuronal surfaces activate signaling pathways leading to synaptotoxicity [22, 23]. While the mechanism on direct neuronal toxicity of PrP^Sc^ has been supported by a large body of experimental data [reviewed by [32, 81]], it does not explain toxicity of prion strains that do not co-localize with neurons. Furthermore, uniform mechanism of PrP^Sc^ toxicity does not elaborate on the question of why brain regions exhibit strikingly different, strain-specific vulnerability to PrP^Sc^ [82, 83]. Recent studies demonstrated that highly infectious prions are not directly neurotoxic [51].

An alternative to the mechanism on direct neurotoxicity of PrP^Sc^ is the hypothesis postulating that reactive microglia and/or astrocytes are involved in synaptic stripping. The mechanisms on synapse elimination by reactive microglia received solid experimental support in several neurodegenerative diseases including Alzheimer's disease and frontotemporal dementia, as well as normal aging [84–87]. Studies on brain development identified key components of the synapse pruning machinery, which include C1q*,* C3 and C4 [88, 89]. Microglia-orchestrated synaptic pruning consists of several steps: tagging of synapses by C1q, then their opsonization by C3, followed by engulfment and phagocytosis of synapses via an interaction with the C3 receptor C3ar1, expressed by microglia. Consistent with this mechanism, several components of a complement cascade including *C1qa, C1qb, C1qc, C3* and *C3ar1* were found to be upregulated in prion-infected mice [50, 90]. Moreover, the sites of proliferation and activation of microglia colocalize with the sites of PrP^Sc^ deposition and neuronal damage [47, 90–92]. The specific mechanism responsible for the microglia-mediated synapse elimination in prion disease remains to be elucidated [93].

The current work tested whether reactive astrocytes associated with prion diseases are neurotoxic or neuroprotective. We found that upon isolation and culturing in vitro*,* astrocytes from prion-infected animals maintained their reactive state characterized by hypertrophic morphology and upregulation of GFAP along with several PAN-, A1-, and A2-specific markers. In addition, several pro-inflammatory genes (*Il6, Il12b, Il33* and *Ccl4)* were upregulated too. At elevated levels, IL-6 is known to trigger a pro-inflammatory pathway linked to neurodegeneration [65]. In previous studies, IL-33 secreted by astrocytes was found to drive microglia-dependent synapse engulfment and elimination [94]. It would be interesting to determine whether reactive astrocytes associated with prion disease boost microglia-dependent synapse-elimination pathways. In addition to upregulation of pro-inflammatory genes, the genes involved in synaptogenic functions (*Thbs1*, *Thbs4*, *Sparcl1*) were downregulated in astrocytes derived from the prion-infected animals. Under normal conditions, products of these genes are necessary for the formation, maturation and stability of synapses and dendritic spines [95, 96]. In particular, astrocyte-secreted thrombospondins promote the formation of new synapses via interaction with their neuronal receptor, calcium channel subunit α2δ-1 [97], whereas SPARCL1 or Hevin, which is also secreted by astrocytes, contribute to excitatory synapse formation [98, 99].

Consistent with the results on gene expression, the experiments on neuronal-astrocyte co-culture revealed that the functions responsible for neuronal growth, spine development and synapse maturation were impaired in astrocytes derived from the prion-infected animals (Fig. 2). To examine whether the deleterious effects of reactive astrocytes were indeed mediated by secreted factors, primary neurons were cultured in the presence of astrocyte-conditioned media. Again, the media conditioned by 22L astrocytes was found to have detrimental effects on viability of neurons and their synaptogenic function (Figs. 3 and 4). In particular, the deleterious effects involved reduction in density and size of the dendritic spine and disintegration of synapses, which was accompanied by reduced expression of pre- and post-synaptic protein synaptophysin and PSD-95, respectively. These changes were reminiscent of the early neuronal abnormalities observed in the brains of prion-infected animals and humans, which involved disintegration of synapses, changed spine morphology followed by loss of spines [72–75, 79, 80].

Several previous studies examined the role of astrocytes in prion diseases [49, 64, 100]. Smith and colleagues examined the role of astrocytic protein kinase R-like endoplasmic reticulum kinase (PERK-P) signaling in prion diseases and found that targeting PERK-P signaling in mice was neuroprotective, prolonging incubation time to the terminal disease [49]. For modelling reactive astrocyte in vitro, Smith et al. purified astrocytes from young pups and treated cultured astrocytes with ER stressors thapsigargin or tunicamycin [49]. Astrocytes stressed in cultures were found to upregulate some PAN reactive markers along with the A1 marker, *C3*. However, strong downregulation of PAN (*Serpina3n*), A1 (*Serping1*), and A2 (*Cd109, Emp1*) markers [49], which are known to be strongly upregulated in prion diseases [21, 50], questions whether astrocytes isolated from pups and treated in vitro with stressors are able to recapitulate the full spectrum of phenotypic changes associated with prion diseases in mature animals. Cronier and co-workers showed that astrocytes infected with prions in cultures accelerated neurodegeneration in astrocyte-neuronal co-cultures, however, they also found that astrocyte-conditioned media was not neurotoxic [100]. The reactive state of astrocytes infected with prions in vitro was not characterized and the extent to which astrocytes cultured in vitro acquire the disease-associated reactive phenotype, in the absence of a brain microenvironment, remains unclear.

To our knowledge, the current study is the first that employed astrocytes purified from prion infected animals for examining their effect on neuronal cultures. Our results illustrate that in the reactive states associated with prion diseases, astrocytes lose their ability to support neuronal growth and formation as well as maintenance of synapses, a reactive phenotype that can be referred to as synaptotoxic. This work suggests that, in addition to the direct synaptic toxicity of PrP^Sc^, a non-cell autonomous astrocyte-dependent mechanism of synaptotoxicity exists in prion disease. In contrast to the mechanisms that rely on direct interaction between PrP^Sc^ and neurons, it is proposed that the astrocyte-dependent mechanism acts via secreted factors, i.e. downregulation of synaptogenic factors and/or upregulation of pro-inflammatory factors. As such, the astrocyte-dependent mechanism might act outside of the immediate sites of reactive astrogliosis. Interestingly, the phenotype of astrocytes derived from prion-infected animals distantly resembled those in normal aging, which is characterized by elevated expression of genes associated with neuroinflammation and synapse elimination [101–103]. The current study does not address the question of whether synaptic loss in vivo occurs due to direct synaptic toxicity of PrP^Sc^ prior to the astrocyte neurotoxic reaction or the other way around. Nevertheless, our previous work on analysis of gene expression revealed that astrocytes respond to prion infection at the pre-clinical stage [50], prior to substantial synaptic loss.

What stimuli do astrocytes in prion infected mice respond to? In vitro, astrocytes can phagocytose and degrade PrP^Sc^ [104]. This suggests that astrocytes have the ability to recognize PrP^Sc^ directly, in the absence of other cell types. It is not clear what molecular features of PrP^Sc^ astrocytes respond to, what receptors are involved in PrP^Sc^ phagocytosis, and whether direct PrP^Sc^-astrocyte interaction activate pro-inflammatory responses in astrocytes. In neurons, lipoprotein receptor-related protein 1 (LR1P), which is also expressed at a high level in astrocytes, was found to be involved in endocytosis of PrP^Sc^ [105]. In microglia, the pro-inflammatory response is triggered directly via interaction of cells with PrP^Sc^, where the sialylation status of N-linked glycans on PrP^Sc^ surface were found to dictate the degree of activation [19, 106]. It remains to be determined whether PrP^Sc^ can directly activate astrocytes too and whether astrocytes have the ability to recognize PrP^Sc^ sialylation status.

What cell types drive chronic neuroinflammation? Toward addressing this question, reactive microglia were isolated from 22L-infected animals. We found that 22L primary microglia cultures exhibited a reactive phenotype characterized by upregulation of proinflammatory genes (*Tnfa, Il1a, Il1b, C1qa, Tlr2, Ccl2, Ccl6, Ccl9. Ccl12, Il10*) and elevated levels of secreted IL-1α, TNF-α and C1q (Additional file 3: Figure S3). In the presence of 22L microglia-conditioned media, primary astrocytes acquired hypertrophic morphology, upregulated PAN- and A1-specific genes, and suppressed genes involved in synaptogenic functions (*Thbs1*, *Thbs4*, *Sparcl1*) (Fig. 5). These phenotypic changes were reminiscent, yet not fully identical to those observed in primary astrocyte cultures isolated from 22L animals. Nevertheless, the result on phenotypic changes in astrocytes induced by microglia-conditions media was in agreement with the hypothesis introduced by Barres and coauthors [107, 108], in which reactive microglia triggers neurotoxic phenotype in astrocytes by secreted factors IL-1α, TNF-α and C1q. In prion diseases, the expression levels of *Il1a, Tnfa* and *C1q* were upregulated [50, 90, 109], yet astrocytes did not exhibit well-defined A1 phenotype, suggesting that the relationship between microglia and astrocytes is complex. Indeed, the recent study demonstrated that the triple (*Il1α*^*−/−*^*, TNFα*^*−/−*^ and *C1q*^*−/−*^) knockout mice infected with prions developed the disease faster in comparison to the control mice [64]. Unexpectedly, only modest changes in reactive phenotype of astrocyte that showed reduced C3 expression was observed in the triple knockout mice relative to the control prion-infected mice, while changes in the phenotype of microglia were substantial upon knocking out *Il1α, TNFα*, *C1q* [64].

In a normal brain, astrocytes exhibit significant region-specific phenotypic heterogeneity [101, 102, 110], yet it is not clear whether the mechanisms of astrocyte activation in response to prion infection is uniform across whole brain. On one hand, region-specific upregulation of microglia- and astrocyte-specific genes follow the same ranking orders with respect to a brain region in animals infected with three prion strains, suggesting that the activation of microglia and astrocytes is tightly coupled to each other [21, 50]. On other hand, astrocyte-specific pathways are activated prior to the microglia-specific pathways [34, 50], raising the possibility that astrocytes respond to prion infection first and dictate the functional state of microglia. Taking into account the results of the current study and the previous work, one can suggest that (i) in addition to the *Il1α/TNFα*/*C1q-*dependent pathway, an alternative mechanisms of astrocyte activation, microglia-dependent or independent, might exist in prion diseases, and (ii) the reactive states of astrocytes and microglia are interdependent.

Studies by Barres and colleagues identified two well-defined astrocyte reactive phenotypes that exhibit distinct transcriptional characteristics and opposite effects on neuronal survival—neurotoxic or A1 and neuroprotective or A2 phenotypes [3, 71]. The concept for classification of astrocytes into A1 and A2 phenotypes was developed using mice treated with lipopolysaccharides (LPS) or subjected to ischemia, respectively [71], conditions that might not induce long-lasting, chronic effects. The A1/A2 astrocyte classification has been useful in establishing the concept of ‘neurotoxic’ or ‘neuroprotective’ astrocytes. However, with growing experimental evidence of multiple reactive states, it now appears that the A1/A2 concept is an over-simplification [111, 112]. Indeed, in animals infected with prions, astrocytes do not display uniform polarization toward A1- or A2-phenotypes, but instead upregulate A1-, A2- and PAN-specific markers [21, 50, 64] along with pro-inflammatory chemokines [113]. In the current study, primary astrocytes isolated from prion infected animals upregulated A1-, A2- and PAN-specific markers as well. It is not known whether in prion diseases such expression pattern arises as a result of the mixture of A1 and A2 astrocytes, existence of multiple activation states in addition to A1 and A2 phenotypes, co-expression A1- and A2- specific markers within individual cells, or all of the above. Moreover, region-specific homeostatic heterogeneity and substantial region-specific variations in astrocytic response to prion infection creates additional challenges to our understanding of their role [47, 114].

In conclusion, the current study provides new experimental evidence in support of the non-cell autonomous mechanisms behind neurotoxicity in prion diseases and demonstrates that the reactive phenotype of astrocytes associated with prion disease is synaptotoxic. In the future studies, it would be interesting to elucidate specific pathways responsible for triggering the synaptotoxicity. Other questions of great interest are whether the reactive, synaptotoxic phenotype is acquired gradually or maintained throughout the course of the disease, and whether it can be reversed.

## Supplementary Information

**Additional file 1: Figure S1.**Generation of adult primary astrocyte cell cultures. ***A***. Schematic illustration of the preparation of primary astrocyte cultures (PACs) from adult C57Bl/6J mouse brain cortices. ***B***. Co-immunostaining of PACs using astrocyte-specific marker (GFAP, green) and microlgia- (Iba1, red), oligodendrocyte- (MBP, red), neuron- (anti-NeuN antibody, red) or second astrocyte-specific marker (S100b, red). Cell nuclei are stained with DAPI (blue). Images are representatives of three independent primary cell cultures, each prepared from an individual animal. ***C***. Analysis of gene expression using qRT-PCR in PACs, normalized by the expression levels in cortical brain homogenates. *Gapdh* was used as a housekeeping gene. Data represent means ± SE (n=3 independent cultures isolated from individual animals), ***p<0.001, **p<0.01 and *p<0.05 (two tailed, unpaired t-test). Scale bar = 50 μm.

**Additional file 2: Figure S2.**Agglomerative hierarchical clustering of brain cortex samples (n=3), acutely isolated astrocytes (n=3) and primary cultured astrocytes (n=5). Primary microglia cultures (n=2) were used as a reference. Adult C57Bl/6J mice (220-283 days old) were used for analysis of astrocyte function related gene expression in bulk tissues and isolated astrocytes and microglia. The scale represents z-score transformed normalized counts for gene transcripts.

**Additional file 3: Figure S3.** Microglia isolated from 22L-infected mice exhibit reactive, proinflammatory phenotype. ***A***. Schematic illustration of the preparation of primary microglia cultures (PMCs) from adult C57Black mouse brain cortex. ***B***. Co-immunostaining of PMCs for microglia (CD11b, green) with astrocytes (GFAP, red), oligodendrocytes (Olig2, red) or neurons (NeuN, red). Cell nuclei are stained with DAPI (blue). Images are representatives of three independent cultures, each from an individual animal. Scale bars= 50 μm. ***C***. Analysis of gene expression in PMCs normalized by the expression levels in cortical brain homogenates using qRT-PCR. ***D***. Analysis of expression of inflammatory genes in 22L-PMCs normalized by the expression levels in CT-PMCs using qRT-PCR. In panels ***C*** and ***D***, *Gapdh* was used as housekeeping gene. ***E***. Representative Western blots and densitometric analysis of Iba1 expression normalized per expression of β-actin in CT-PACs and 22L-PACs. ***F***. Analysis of secreted levels of TNF-a, IL-1α and C1q in media conditioned by CT-PMCs and 22L-PMCs. In ***C***, ***D*** and ***E***, data represent means ± SE, n=3 independent cultures isolated from individual animals, each analyzed in triplicates, ***p<0.001, **p<0.01 and *p<0.05 and ‘ns’ non-significant (two tailed, unpaired student t-test).

## Data Availability

All data generated or analyzed during this study are included in this published article and its supplementary information file.

## Abbreviations

22L: Mouse adapted prion strain
22L-PAC: Primary astrocyte culture derived from 22L animals
CT-PAC: Primary astrocyte culture derived from age-matched control animals
ACM: Astrocyte-conditioned media
22L-ACM: Astrocyte-conditioned media from primary astrocyte culture derived from 22L animals
CT-ACM: Astrocyte-conditioned media from primary astrocyte culture derived from age-matched control animals
22L-PMC: Primary microglia cultures derived from 22L animals
CT-PMC: Primary microglia cultures derived from 22L age-matched control animals
GFAP: Glial fibrillary acidic protein
GPI: Glycosylphosphatidylinositol
PrP^C^: Normal, cel;lular isoform of the prion protein
PrP^Sc^: Infectious, disease-associated, pathogenic form of the prion protein
