# Supplementary figures and images for: Non-cell autonomous astrocyte-mediated neuronal toxicity in prion diseases
Source: Acta Neuropathol Commun. 2021 Feb 5;9:22. doi: 10.1186/s40478-021-01123-8 (PMC7866439; doi:10.1186/s40478-021-01123-8)

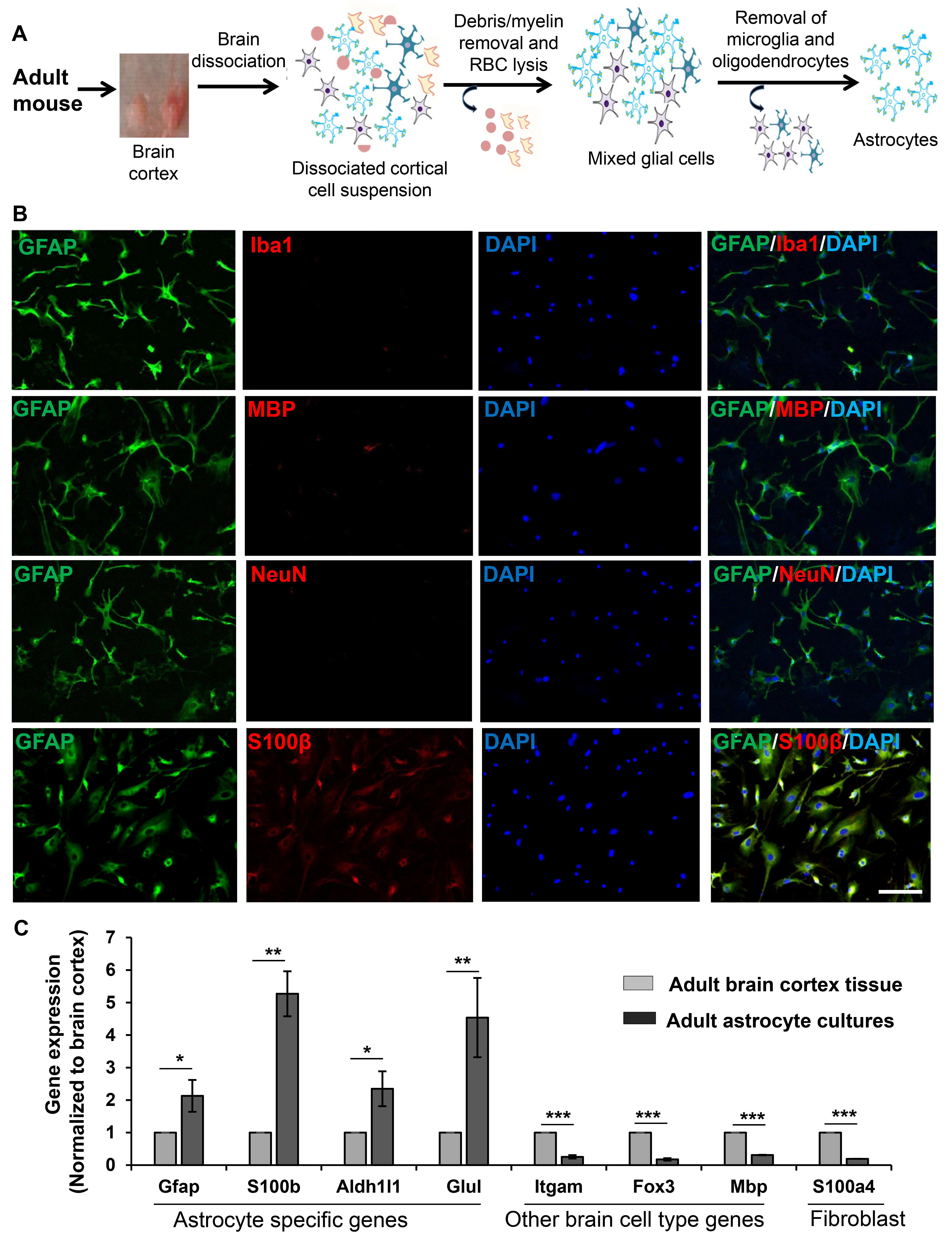

Supplement: Supplementary file 1 — Additional file 1: Figure S1.Generation of adult primary astrocyte cell cultures. A. Schematic illustration of the preparation of primary astrocyte cultures (PACs) from adult C57Bl/6J mouse brain cortices. B. Co-immunostaining of PACs using astrocyte-specific marker (GFAP, green) and microlgia- (Iba1, red), oligodendrocyte- (MBP, red), neuron- (anti-NeuN antibody, red) or second astrocyte-specific marker (S100b, red). Cell nuclei are stained with DAPI (blue). Images are representatives of three independent primary cell cultures, each prepared from an individual animal. C. Analysis of gene expression using qRT-PCR in PACs, normalized by the expression levels in cortical brain homogenates. Gapdh was used as a housekeeping gene. Data represent means ± SE (n=3 independent cultures isolated from individual animals), ***p<0.001, **p<0.01 and *p<0.05 (two tailed, unpaired t-test). Scale bar = 50 μm. [file 40478_2021_1123_MOESM1_ESM.tif]

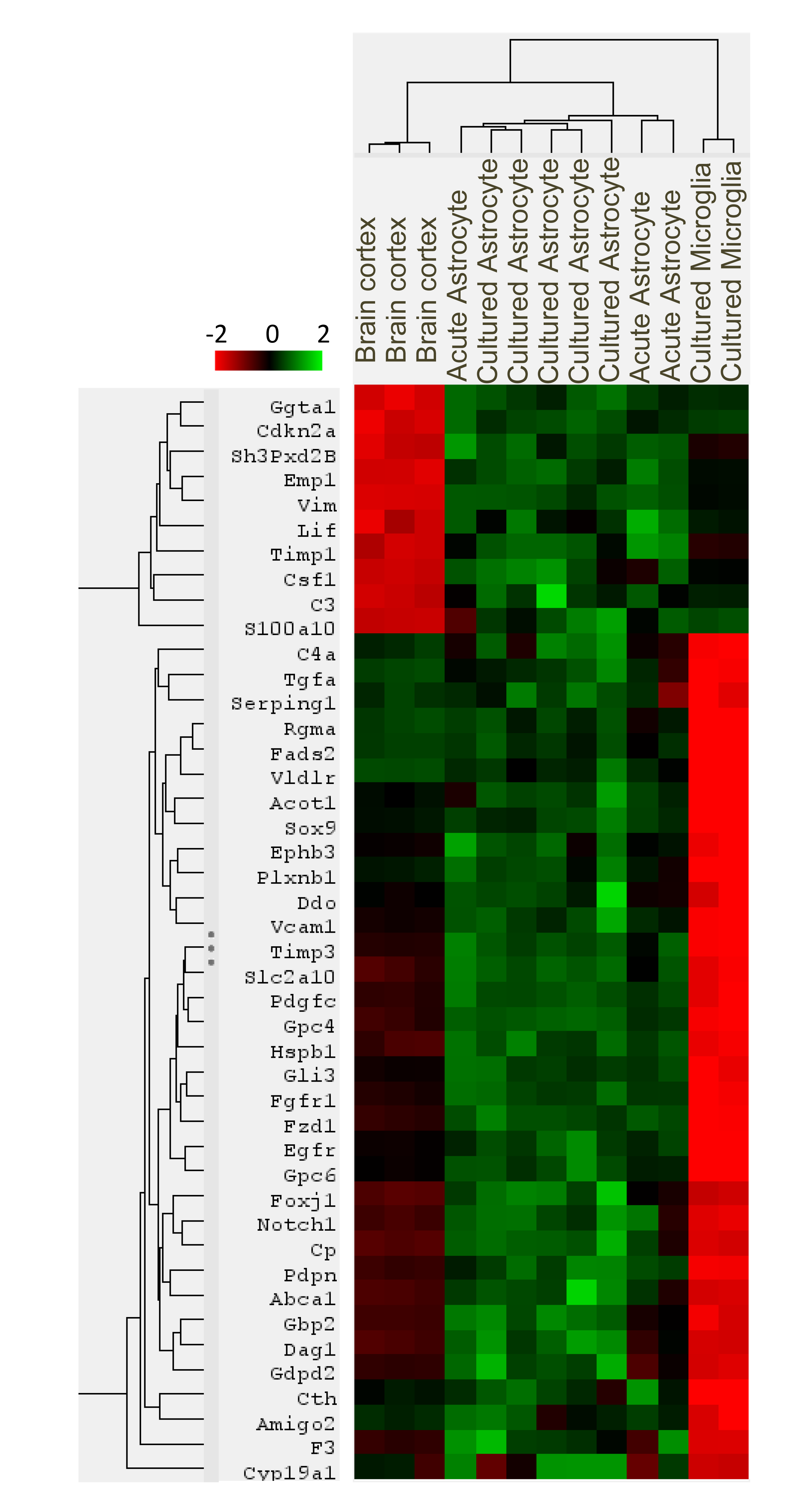

Supplement: Supplementary file 2 — Additional file 2: Figure S2.Agglomerative hierarchical clustering of brain cortex samples (n=3), acutely isolated astrocytes (n=3) and primary cultured astrocytes (n=5). Primary microglia cultures (n=2) were used as a reference. Adult C57Bl/6J mice (220-283 days old) were used for analysis of astrocyte function related gene expression in bulk tissues and isolated astrocytes and microglia. The scale represents z-score transformed normalized counts for gene transcripts. [file 40478_2021_1123_MOESM2_ESM.tif]

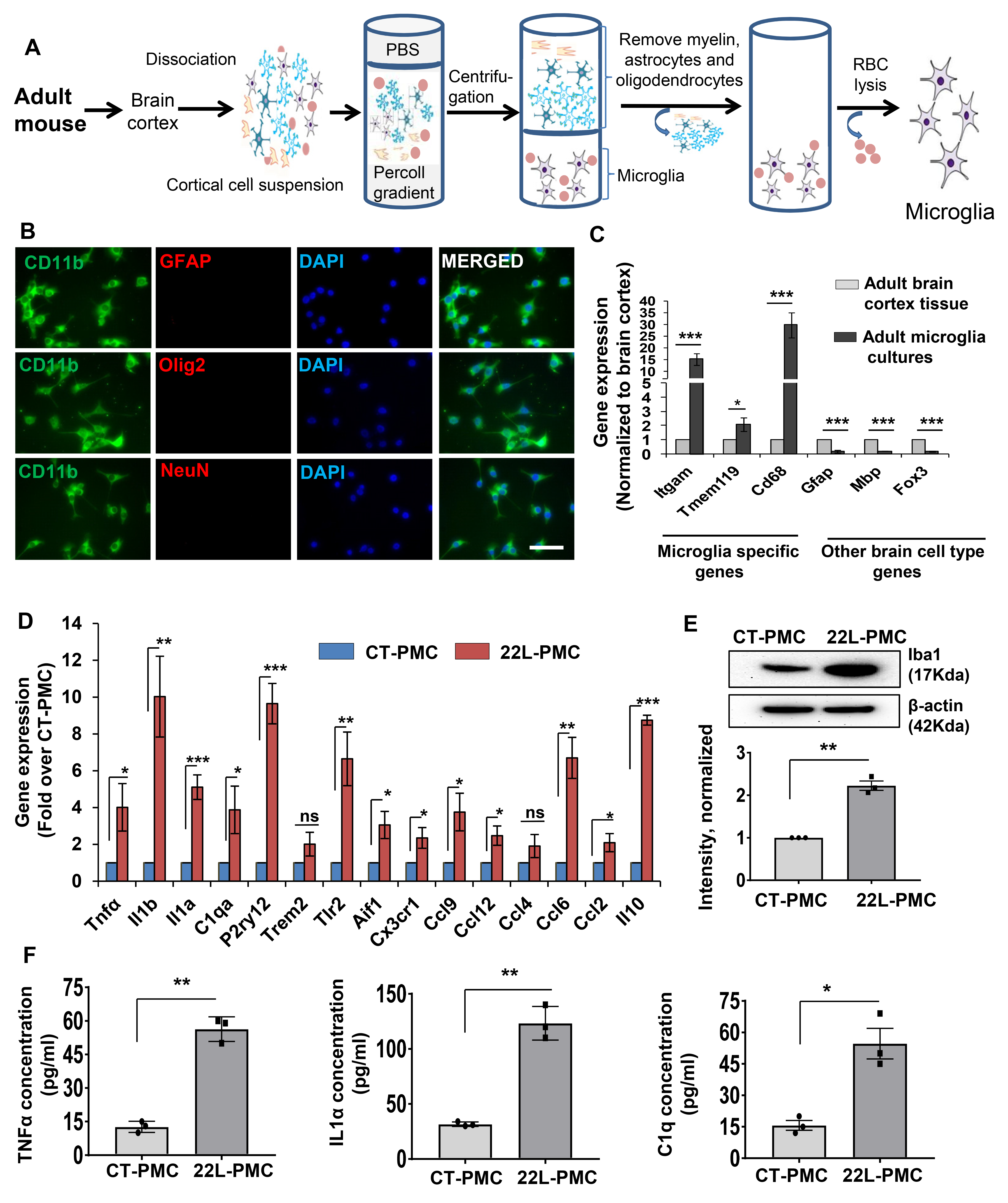

Supplement: Supplementary file 3 — Additional file 3: Figure S3. Microglia isolated from 22L-infected mice exhibit reactive, proinflammatory phenotype. A. Schematic illustration of the preparation of primary microglia cultures (PMCs) from adult C57Black mouse brain cortex. B. Co-immunostaining of PMCs for microglia (CD11b, green) with astrocytes (GFAP, red), oligodendrocytes (Olig2, red) or neurons (NeuN, red). Cell nuclei are stained with DAPI (blue). Images are representatives of three independent cultures, each from an individual animal. Scale bars= 50 μm. C. Analysis of gene expression in PMCs normalized by the expression levels in cortical brain homogenates using qRT-PCR. D. Analysis of expression of inflammatory genes in 22L-PMCs normalized by the expression levels in CT-PMCs using qRT-PCR. In panels C and D, Gapdh was used as housekeeping gene. E. Representative Western blots and densitometric analysis of Iba1 expression normalized per expression of β-actin in CT-PACs and 22L-PACs. F. Analysis of secreted levels of TNF-a, IL-1α and C1q in media conditioned by CT-PMCs and 22L-PMCs. In C, D and E, data represent means ± SE, n=3 independent cultures isolated from individual animals, each analyzed in triplicates, ***p<0.001, **p<0.01 and *p<0.05 and ‘ns’ non-significant (two tailed, unpaired student t-test). [file 40478_2021_1123_MOESM3_ESM.tif]
